# Fast water transport and ionic sieving in ultrathin stacked nanoporous 2D membranes

**DOI:** 10.1093/nsr/nwae482

**Published:** 2025-01-06

**Authors:** Jingfeng Wang, Xiaoming Zhang, Zehua Yu, Yuyan Gao, Qingqing Lu, Chao Ma, Kang Liu, Quan Yuan, Yanbing Yang

**Affiliations:** College of Chemistry and Molecular Sciences, Key Laboratory of Biomedical Polymers of Ministry of Education, School of Power and Mechanical Engineering, Institute of Molecular Medicine, Renmin Hospital of Wuhan University, School of Microelectronics, Wuhan University, Wuhan 430072, China; College of Chemistry and Molecular Sciences, Key Laboratory of Biomedical Polymers of Ministry of Education, School of Power and Mechanical Engineering, Institute of Molecular Medicine, Renmin Hospital of Wuhan University, School of Microelectronics, Wuhan University, Wuhan 430072, China; College of Chemistry and Molecular Sciences, Key Laboratory of Biomedical Polymers of Ministry of Education, School of Power and Mechanical Engineering, Institute of Molecular Medicine, Renmin Hospital of Wuhan University, School of Microelectronics, Wuhan University, Wuhan 430072, China; Department of Engineering Science and Mechanics, The Pennsylvania State University, University Park, PA 16802, USA; College of Chemistry and Molecular Sciences, Key Laboratory of Biomedical Polymers of Ministry of Education, School of Power and Mechanical Engineering, Institute of Molecular Medicine, Renmin Hospital of Wuhan University, School of Microelectronics, Wuhan University, Wuhan 430072, China; Molecular Science and Biomedicine Laboratory (MBL), State Key Laboratory of Chemo/Biosensing and Chemometrics, College of Chemistry and Chemical Engineering, College of Materials Science and Engineering, Hunan University, Changsha 410082, China; College of Chemistry and Molecular Sciences, Key Laboratory of Biomedical Polymers of Ministry of Education, School of Power and Mechanical Engineering, Institute of Molecular Medicine, Renmin Hospital of Wuhan University, School of Microelectronics, Wuhan University, Wuhan 430072, China; College of Chemistry and Molecular Sciences, Key Laboratory of Biomedical Polymers of Ministry of Education, School of Power and Mechanical Engineering, Institute of Molecular Medicine, Renmin Hospital of Wuhan University, School of Microelectronics, Wuhan University, Wuhan 430072, China; Molecular Science and Biomedicine Laboratory (MBL), State Key Laboratory of Chemo/Biosensing and Chemometrics, College of Chemistry and Chemical Engineering, College of Materials Science and Engineering, Hunan University, Changsha 410082, China; College of Chemistry and Molecular Sciences, Key Laboratory of Biomedical Polymers of Ministry of Education, School of Power and Mechanical Engineering, Institute of Molecular Medicine, Renmin Hospital of Wuhan University, School of Microelectronics, Wuhan University, Wuhan 430072, China

**Keywords:** stacked structure, membranes, layer, water transport, ionic sieving

## Abstract

Atomically thin nanoporous 2D membranes, featuring unique sieving characteristics for molecules and ions, have significant potential for seawater desalination. However, they face a common trade-off between permeability and selectivity. Here, we report an ultrathin stacked nanoporous graphene membrane (SNGM) created by layering atomically thin graphene nanomesh. This design achieves highly efficient and selective sieving of water molecules and ions. The SNGMs showcase in-plane nanopores for optimal size-exclusive water input and output, and interlayer 2D nanochannels between adjacent graphene nanomesh membranes for rapid water transport and precise ion/molecular sieving. The resulting SNGMs effectively address the trade-off between water permeability and ion selectivity in conventional desalination membranes, delivering a water permeability of ∼ 1–2 orders of magnitude higher than that of commercial membranes, while maintaining a comparable ion rejection ratio (>95% for NaCl). This advance marks a significant leap forward in adopting 2D nanoporous membranes for desalination technology.

## Teaser

A stacked nanoporous graphene membrane assembled by a few layers of atomically thin graphene nanomesh was constructed, achieving highly efficient sieving of water molecules/ions and resolving the permeability-selectivity trade-off in conventional membranes.

## INTRODUCTION

Atomically thin 2D materials have been considered as ideal building blocks for constructing high-performance membranes for molecules and ionic sieving because of their unique physical and chemical stability, and have profound implications for water desalination applications [1–6]. Nanoporous 2D membranes mainly consist of atomically thin monolayer nanoporous membranes and lamellar nanoporous membranes [7–10]. The atomically thin monolayer nanoporous membranes were obtained by introducing nanopores in 2D materials, where the in-plane nanopores play a vital role in molecular sieving [11–14]. On the other hand, the lamellar nanoporous membranes assembled by small 2D nanosheets offer an alternative strategy to exploit the interlayer 2D nanochannels for molecular and ionic transport [15–21]. In either case, these nanoporous 2D membranes suffer a common trade-off between permeability and selectivity, limiting their applications in efficient water desalination [22–26].

The efficient sieving relies on the interaction between molecules/ions and nanochannels in the membranes [27,28]. Atomically thin nanoporous 2D membranes offer ultralow water transport resistance because of their unique single-atomic thickness characteristics [29–31]. Water molecules and ions could transport across the in-plane nanopores in the atomically thin membranes rapidly due to the negligible interaction between molecules/ions and membranes [32–35]. Thus, atomically thin nanoporous 2D membranes generally feature high water permeability and low ion selectivity [36,37]. Lamellar 2D membranes formed by assembling small 2D nanosheets with oxygen-, fluorine-containing functional groups provide tortuous transport pathways for ions, enhancing the interactions between ions and membrane to enable improved ion selectivity [38], but substantially reducing water permeability resulting from the increased tortuosity and interactions [39,40].

**Figure 1.**
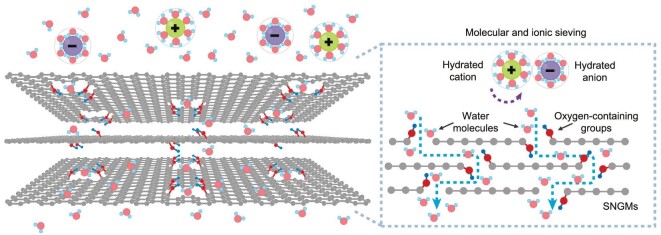
Schematic illustration of the SNGMs for efficient molecular sieving. The dashed box shows the selective sieving mechanism of the SNGMs for molecules and ions.

Here, we report the design of a stacked nanoporous graphene membrane (SNGM) assembled by a few layers of atomically thin large-area graphene nanomesh (Fig. 1). The constructed SNGMs feature high permeability and selectivity for efficient transport of water molecules while blocking ions. The nanopores in the atomically thin graphene nanomesh provide ultrashort nanochannels for water molecule transport, and the oxygen-containing groups at the edge of nanopores act as spacers to keep interlayer 2D nanochannels open for water molecule transport. The absence of oxygen-containing groups in the plane domains of graphene nanomesh offers a smooth 2D nanochannel for the achievement of decreased interaction between water molecules and 2D nanochannels, thus improving water permeability. With the size exclusion of the in-plane nanopores and the gating effect of the interlayer 2D nanochannels to synergistically tailor the molecular and ionic sieving properties, the SNGMs exhibit a high-water permeability of 77.8 L m^–2^ h^–1^ bar^–1^, and an excellent monovalent salt ions rejection ratio (up to 95.2%). The SNGMs offer an efficient platform for probing the underlying molecules/ions transport behavior in nano-confined spaces, with significant potential to effectively address the common permeability–selectivity trade-off in conventional nanoporous membranes.

## RESULTS AND DISCUSSION

### Design and structural characterizations of SNGMs

**Figure 2.**
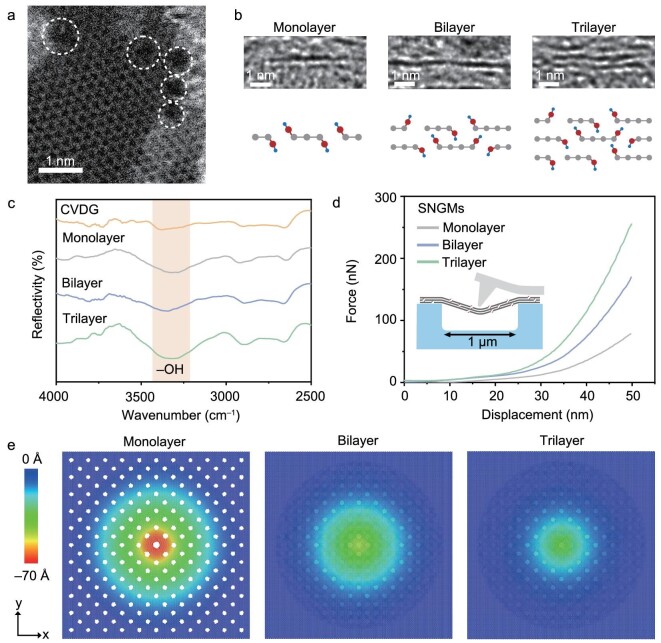
Structural and mechanical performance characterizations. (a) Aberration-corrected STEM image of the monolayer graphene nanomesh. The white dashed circles highlight the nanopores present in the monolayer graphene nanomesh. (b) Cross-sectional high-resolution TEM images of the monolayer graphene nanomesh, and SNGMs assembled by bilayer and trilayer graphene nanomesh. (c) FTIR spectra of pristine chemical vapor deposition graphene (CVDG), monolayer graphene nanomesh, and SNGMs assembled by bilayer and trilayer graphene nanomesh. (d) Load-displacement curves of monolayer graphene nanomesh, and SNGMs assembled by bilayer and trilayer graphene nanomesh. Inset: schematic illustration of the nanoindentation tests on suspended SNGMs. (e) The spatial distribution of the SNGMs at 100 nN force in MD simulations. The data were derived from MD simulations.

The SNGMs were formed by sequential stacking of atomically thin graphene nanomesh prepared by a mesoporous template combined with O_2_ plasma exposure (Fig. S1). An aberration-corrected scanning transmission electron microscope (STEM) image of the pristine monolayer graphene shows a typical hexagonal honeycomb lattice structure composed of carbon atoms (Fig. S2). By comparison, the STEM image of the SNGM shows the presence of sub-nanometer pores, with an average pore size of ∼5 Å and a pore density of ∼3.0 × 10^12^ cm^−2^ (Fig. 2a; Figs S3 and S4). This pore size matches well with the optimized sub-nanometer pores for water molecules (∼3 Å) transport while effectively rejecting hydrated ions (>7 Å). The lamellar structure and layer numbers were observed in the high-resolution TEM images of the cross-section of SNGMs (Fig. 2b). The interlayer spacing of 2D nanochannel in the SNGMs is measured to be ∼4 Å, which is similar to previously reported 2D interlayer space that allows fast transport of one monolayer of water molecules [41]. The TEM characterizations suggest that the designed SNGMs possess optimized in-plane nanopores and 2D nanochannels for ultrafast water molecules transport and ion sieving.

Raman spectroscopy was performed to analyze the number of layers and surface defects of the SNGMs (Fig. S5). The number of layers was identified according to the intensity ratio between the G (*I*_G_) and 2D (*I*_2D_) peaks (*I*_G_/*I*_2D_), and the intensity ratio between the D (*I*_D_) and G (*I*_G_) peaks (*I*_D_/*I*_G_) was utilized to investigate the surface defects, respectively. As shown in Fig. S5, with the increase of the layer number from monolayer to trilayer, the *I*_G_/*I*_2D_ increases from 0.5 to 1.2 and the *I*_D_/*I*_G_ increases from 0.2 to 0.5, suggesting that the sequential stacking of graphene nanomesh increases the defects in the SNGMs. Fourier-transformed infrared (FTIR) spectra and X-ray photoelectron spectra (XPS) characterizations show a strong characteristic absorption peak corresponding to hydroxyl groups (broad peak between ∼3200 and ∼3500 cm^–1^) (Fig. 2c, Fig. S6 and Table S1), mainly attributed to oxygen-containing groups at the pore edges of SNGMs after O_2_ plasma treatment. The studies suggest that the SNGMs with high-density sub-nanopores and abundant hydroxyl groups were successfully constructed.

The mechanical properties of the SNGMs were investigated by nanoindentation in an atomic force microscope (AFM) and molecular dynamics (MD) simulations. The SNGMs were suspended on a SiN substrate with arrays of apertures (diameter 1.0 μm) to construct a micro-testing system (Fig. S7). The typical force–displacement curves of the SNGMs assembled with different layers of graphene nanomesh were recorded by the AFM. As indicated in Fig. 2d, at the same indentation depth, the indentation forces of the SNGMs increase with the increase of stacked layer numbers of graphene nanomesh. Specifically, to achieve an indentation depth of 50 nm, the indentation force for the SNGMs assembled by bilayer graphene nanomesh is 848.6 nN, which is higher than that of monolayer graphene nanomesh. As the stacking layers increased from bilayer to trilayer, the required indentation force for the SNGMs increased by 31.9%. MD simulations reveal that the indentation force at the same indentation depth increases with an increasing number of graphene nanomesh layers (Figs S8 and S9), consistent with the nanoindentation tests (Fig. S10). The increase in force indicated an enhancement of resistance to external loads due to the increased interlayer interactions. For the monolayer graphene nanomesh, the in-plane strain distribution is highly localized at the geometry center of the corresponding layer, which is directly underneath the indenter tip. The localization of in-plane strain distribution is gradually weakened with an increasing number of graphene nanomesh layers (Fig. 2e and Figs S11-S13). Strain analysis revealed that strain shifting away from geometry centers is more prominent with increased interlayer interactions, thus resulting in a reduced strain concentration near the geometry center. These analyses indicate that the interlayer interactions including van der Waals and hydrogen bonding interactions in the SNGMs effectively improve the mechanical strength and structural stability of the SNGMs. The improved structural stability is beneficial to avoid the diminishing of ion selectivity caused by the rupture of SNGMs in the molecules/ions sieving processes.

### Transport behavior of water molecules within SNGMs

**Figure 3.**
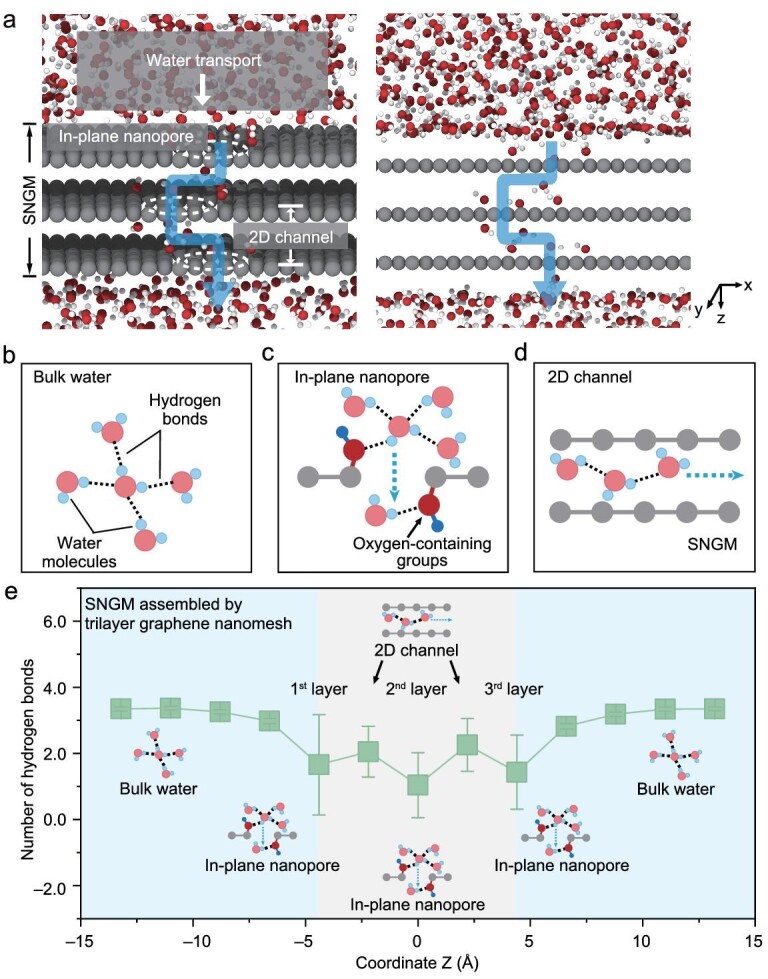
Water transport behavior investigations. (a) A MD simulation snapshot of the water transport in the SNGMs assembled by trilayer graphene nanomesh. The z-direction indicated by the black arrow is perpendicular to the membrane. (b–d) Schematic illustrating the different hydrogen bonds patterns for water molecules in the bulk state (b), in-plane nanopore (c) and 2D channel (d) inside the SNGMs. (e) Hydrogen bonds distribution among water molecules in the SNGMs assembled by trilayer graphene nanomesh. Inset: schematic illustrating the state of water through the in-plane nanopore and 2D nanochannels.

MD simulations were conducted to explore the transport properties of water molecules within the SNGMs (Fig. 3a, Figs S14 and S15). Figure 3a shows the transmembrane transport pathways of water molecules in the SNGMs. Specifically, water molecules sequentially passed through the in-plane nanopores of the first-layer graphene nanomesh, and then entered the interlayer 2D nanochannels formed between the neighboring graphene nanomesh and finally flowed out of the SNGMs through the in-plane nanopores of the bottom-layer graphene nanomesh. The specific transport behaviors of water molecules in the in-plane nanopores and 2D nanochannels of the SNGMs were further analyzed (Fig. S16). The dynamic hydrogen bonds among water molecules in the bulk and between water molecules and oxygen-containing groups in the SNGMs (Fig. 3b–d) play a critical role in water molecule transport. When the water molecules transport from the bulk water into the in-plane nanopores, the average number of hydroxy bonds among water molecules dropped from 3.4 to 1.5, indicating that there were hydrogen bonds between water molecules and hydroxyl groups at the pore edge of graphene nanomesh (Fig. 3e, Figs S17 and S18). These hydrogen bonds increase the interaction between water molecules and the nanopores and lower the barrier for water molecules to enter the nanopores. This enhanced transport property is in agreement with previous reports that hydroxyl groups can attract water molecules into nanopores [42].

When water molecules transport from the in-plane nanopores to 2D nanochannels in the SNGMs, the average number of hydroxy bonds among water molecules increases to ∼2.1, suggesting that there are interactions between water molecules within the 2D nanochannels, but remains much smaller than that of bulk water (3.4) (Fig. 3e and Figs S19-S22). Water molecules with a smaller number of hydrogen bonds usually lead to less viscous fluids, as the intermolecular forces are weaker under such conditions, causing less resistance to water flow [43]. This weak interaction allows the fast transport of water molecules (∼3 Å) inside the interlayer 2D nanochannels (interlayer spacing ∼4 Å) in the form of 2D monolayer, which is similar to the molecular superfluidity phenomenon in hydrophobic nano-confined spaces [34,44].

Together, the in-plane nanopore in the SNGMs is favorable for water molecules to enter the membranes through hydrogen bond interactions, and the weak interaction between the 2D nanochannels and water molecules allows ultrafast water permeation. This unique water molecule transport behavior is beneficial for improving the water permeability.

### Water molecules’ transport velocity and water permeability

**Figure 4.**
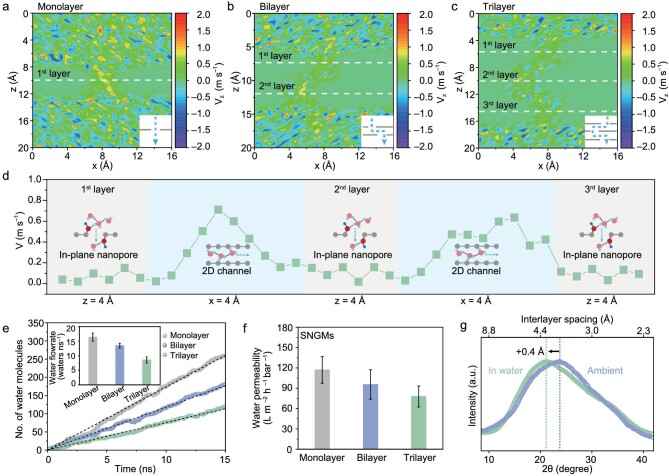
Water permeability evaluations. (a–d) Time-averaged water molecules transport velocity in the monolayer graphene nanomesh (a) and SNGMs assembled by bilayer graphene nanomesh (b) and trilayer graphene nanomesh (c and d) (x-z plane). (e) The transmembrane transport number of water molecules and water permeability of the SNGMs assembled with different layers of graphene nanomesh as a function of simulation time (t). Inset: the calculated water flow rate of the SNGMs. (f) Measured water permeability of the SNGMs assembled with different layers of graphene nanomesh. The water permeability was measured with a cross-flow filtration apparatus. (g) XRD showing the shifts of the peak corresponding to the interlayer distance of the 2D nanochannels in the SNGMs before and after 48 hours of tests in a water environment.

We further evaluated the water molecule transport velocities through the SNGMs by MD simulations. The transport velocities of water molecules through the in-plane nanopore and 2D nanochannels of the SNGMs with different layers of graphene nanomesh were calculated and are plotted in Fig. 4a–c. The transport velocities of water molecules slightly decrease with the increasing graphene nanomesh layers. An average transport velocity of 0.09 m s^−1^ is achieved through the in-plane nanopores, while the water transport velocity through the 2D nanochannels is considerably faster (0.35 m s^−1^) (Fig. 4d and Fig. S23). Thus, the water transport through the nanopore dictates the overall transmembrane transport. We have further evaluated the number of water molecules transported through the SNGMs and the corresponding water permeability with a different number of layers stacked graphene nanomesh (Fig. 4e, Figs S24 and S25). Overall, monolayer graphene nanomesh shows a maximum water flow rate of 16 waters ns^−1^ due to the minimum transport resistance [45,46]. The sequential stacking of graphene nanomesh increases the water molecules transport pathway in the SNGMs and reduces the flow rate (Fig. 4e, inset); the water flow rate still maintains >8 waters ns^−1^ with three layers of stacked graphene nanomesh.

To further explore the water transport performance of the SNGMs, we constructed a reverse osmosis (RO) cross-flow filtration apparatus to experimentally determine the water permeability of the membrane (Figs S26-S32). The water permeability of the SNGMs assembled with different layers of graphene nanomesh was maintained at 77.8–117.0 L m^–2^ h^–1^ bar^–1^ for pure water, and the measured values agree well with the theoretically calculated trend (Fig. 4f and Fig. S33). The above results demonstrate that a combination of high-density in-plane nanopores and ultrafast 2D nanochannels in the SNGMs ensures low transport resistance for efficient water molecule transport.

X-ray diffraction (XRD) studies of the SNGMs indicate that interlayer spacing only increases from 3.8 to 4.2 Å (Fig. 4g), suggesting a moderate swelling in the water environment [47], which is favorable for water transport and maintaining the structural stability of the SNGMs. The excellent structural stability of SNGMs is mainly attributed to the decreased number of oxygen-containing groups in the 2D nanochannels that avoids the insertion of water molecules into the interlayer nanochannels and the swelling of the SNGMs. The above results indicate that the constructed SNGMs feature ultrahigh water molecule transport velocity and high water permeability, and could maintain excellent structural stability after long-term operation.

### Ionic sieving performance evaluations

**Figure 5.**
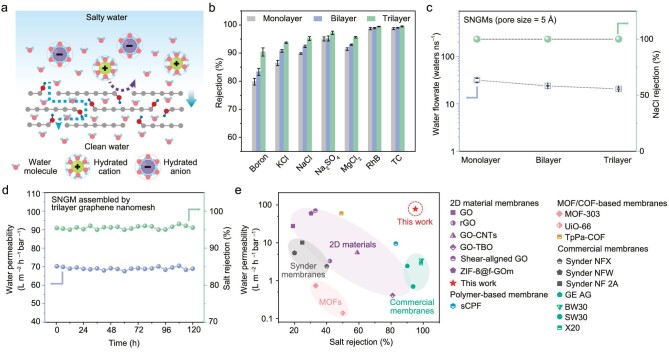
Evaluation of molecular and ionic sieving performance. (a) Schematic of the SNGMs for molecular and ionic sieving. (b) Rejection ratio of the SNGMs for KCl, NaCl, Na_2_SO_4_, MgCl_2_, boron, rhodamine B (RhB) and tetracycline antibiotic (TC). The error bars indicate the standard deviation of the data acquired from three individual membranes. (c) The calculated water flow rate in NaCl solutions and NaCl rejection of the SNGMs with the pore size of 5 Å in MD simulations. (d) Long-term stability of the SNGMs assembled by trilayer graphene nanomesh conducted under cross-flow conditions. (e) Comparison of the water permeability and ions rejection performance of the SNGMs assembled with trilayer graphene nanomesh with state-of-the-art membranes reported in the literature. GO, graphene oxide; rGO, reduced graphene oxide; MOF, metal–organic framework; COF, covalent–organic framework; CPF, conjugated-polymer framework; Synder NFX and Synder NFW are commercial membranes.

A cross-flow RO filtration apparatus was constructed to investigate the molecular and ionic sieving performance of the SNGMs (Fig. 5a, Figs S26 and S27). In the chamber of the RO filtration apparatus, the direction of water permeation direction is perpendicular to the direction of water flow, thus the water will be driven by the external pumping pressure and hydrostatic pressure generated by the gravity of water together to overcome the osmotic pressure and pass through the membrane into the fresh water tank [48–50]. The salt ions rejection performances of the SNGMs assembled with different layers of graphene nanomesh were all determined to be >85% for monovalent/divalent salt ions (Fig. 5b). The measured salt rejections of monolayer membrane are 89.3% for Na^+^ (hydrated diameter ∼7.2 Å) and 86.5% for K^+^ (hydrated diameter ∼6.6 Å), respectively. The slight ion leakage is attributed to the presence of a small fraction of relatively large nanopores in the monolayer graphene nanomesh and intrinsic defects or cracks formed during the graphene growth or transfer processes.

The lamellar structure with stacked layer can effectively avoid the leakage through intrinsic defects or cracks in the monolayer SNGMs membrane. Additionally, the inclusion of interlayer 2D nanochannels in the SNGMs increases the interaction between salt ions and the membrane. The ions rejection performance of the SNGMs gradually increases with the stacking layers of graphene nanomesh. The rejection ratio of the bilayer and trilayer SNGMs exceeds 95.2% and 95.6%, respectively. Monolayer graphene nanomesh and the bilayer SNGMs exhibit a rejection ratio of 79.8% and 83.3% for boron (∼5.0 Å), respectively. Notably, the trilayer SNGMs exhibit a rejection ratio of >90.4% for boron. The measured selectivity of the SNGMs follows the order of Na_2_SO_4 _> MgCl_2 _> NaCl > KCl > boron, which was positively correlated to the hydration diameter of ions. Additionally, the SNGMs show a high rejection ratio for small charged or neutral molecules (99.4% and 99.5% for rhodamine B and tetracycline antibiotic molecules, respectively) with solvated diameters of ∼10 Å (Fig. S34). This excellent sieving performance for organic molecules is difficult to achieve in typical commercial polymer membranes with poor chemical stability. It is worth mentioning that the water permeability of the SNGMs assembled with different layers of graphene nanomesh maintains 66.0–95.3 L m^–2^ h^–1^ bar^–1^ in the salt solution or organic molecular solution compared with that of pure water (77.8–117.0 L m^–2^ h^–1^ bar^–1^) (Figs S35-S37) and that the SNGMs exhibited an extremely low adsorption percentage (<0.5%; Fig. S38). Moreover, the water permeability of the SNGMs only decreased by 33.5% because of the increase of graphene nanomesh stacking layers, suggesting that the proposed lamellar structure improves the ionic sieving selectivity while retaining a high-water permeability.

A MD simulation model was constructed to explore the molecular and ionic sieving mechanism of the SNGMs. Figure 5c and Fig. S39 illustrate the sieving performance of SNGMs stacked by graphene nanomesh with pore sizes of 5 and 8 Å, assuming a fixed interlayer spacing of 4 Å. A 100% NaCl rejection performance is observed in monolayer graphene nanomesh with a pore size of 5 Å that is smaller than the diameter of hydration Na^+^ ions (>7 Å) (Fig. 5c), which reduces to 96.7% in an 8 Å pore size graphene nanomesh. This result indicates that the size exclusion effect of in-plane nanopores can effectively prevent salt ions from passing through the membrane. As the stacking of graphene nanomesh increased from monolayer to bilayer or trilayer, the SNGMs with in-plane pore sizes of 5 or 8 Å all show a prominent salt rejection of 100%, suggesting that the interlayer spacing in stacked 2D membranes can effectively improve salt rejection performance. The water molecule or ion transmembrane transport number decreases with the increase of stacked graphene nanomesh layers, which is consistent with the experimental results (Fig. 4f). The salt rejection showed a slight decrease when the pH was varied from 7 to 3 (the protonation of oxygen-containing groups at low pH values) (Fig. S40), suggesting that the surface-charge effect was involved in the sieving process. The above results indicate that the size exclusion effect of in-plane nanopores and interlayer 2D nanochannels and the gating effect in 2D nanochannels play a synergetic role in the molecular/ion sieving [51–56]. The fundamental reason for this is that the stacking of graphene nanomesh includes enormous 2D nanochannels in the SNGMs, resulting in increased ion transport pathway and interaction.

The long-term stability of the SNGMs was investigated under the same cross-flow velocity and hydraulic pressure conditions (Fig. 5d, Figs S41 and S42). The water permeability of the monolayer SNGM shows a slight increase from 98.4 to 120.3 L m^–2^ h^–1^ bar^–1^ after 90 hours of measurement, while the ions rejection ratio decreased by 10.0%. By comparison, the bilayer and trilayer SNGMs retain relatively stable water permeability (69.0–77.3 L m^–2^ h^–1^ bar^–1^) and salt rejection ratio (92.4–95.5%). Moreover, our studies of the trilayer SNGMs show that they maintain stable water permeability and salt rejection, even after several cycles of desalination/drying (Fig. S43). The increased water permeability of the monolayer SNGM is due to the intrinsic defects or cracks in the membrane being enlarged gradually after continuous operation and more ions being allowed to penetrate through the membrane. By contrast, the lamellar structure improves the mechanical strength of the SNGMs and allows the intrinsic defects in atomically thin graphene nanomesh to be shielded, ensuring long-term stability in water permeability and maintenance of the ions rejection performance. Similar water permeability and ions rejection performance were observed in more than 100 SNGMs. The SNGMs exhibit high resistance to bacterial attachment compared with the commercial cellulose triacetate membrane after long-term operation. The excellent anti-biofouling characteristic is mainly attributed to the high hydrophilicity of the SNGMs that hinders the attachment of bacteria (Fig. S44).

Compared with commercial RO membranes, the SNGMs exhibit ∼1–2 orders of magnitude higher water permeability (Fig. 5e and Table S2), while achieving a comparable ion rejection ratio (>95% for NaCl), which is higher than that of most graphene- or graphene oxide-based membranes, metal-organic framework-, covalent–organic framework- or conjugated-polymer framework-based membranes. These results demonstrate that the SNGMs exhibit a combination of water permeability, ion rejection ratio and structural stability, and thus effectively address the common trade-off between water permeability and ion selectivity in conventional desalination membranes.

## CONCLUSION

We have constructed an SNGM consisting of a few layers of atomically thin graphene nanomesh featuring excellent molecular and ionic sieving performance. The van der Waals and hydrogen bonding interactions in the SNGMs effectively shield the defects in monolayer graphene nanomesh and result in greatly improved ion/molecular sieving properties and overall structural stability. The hydroxy groups in the in-plane nanopores could attract water molecules into the membranes, and the interlayer 2D nanochannels with smooth surfaces allow the ultrafast transport of water molecules in the form of monolayers, resulting in an optimized water permeability ∼1–2 orders of magnitude higher than that of commercial membranes. The size exclusion effect of in-plane nanopores and the gating effect of 2D nanochannels increase the interaction between ions and SNGMs, achieving precise ion sieving with selectivity of up to 97.2%. The SNGMs design addresses the fundamental permeability–selectivity trade-off in nanoporous membranes, thus promising attractive molecular/ionic separation membranes for the chemical, energy and biomedicine industries.

## Supplementary Material

nwae482_Supplemental_Files

## References

[1] Wang J, Cheng C, Zheng X et al. Cascaded compression of size distribution of nanopores in monolayer graphene. Nature 2023; 623: 956–63. 10.1038/s41586-023-06689-y 38030784

[2] Chen G, Chen C, Guo Y et al. Solid-solvent processing of ultrathin, highly loaded mixed-matrix membrane for gas separation. Science 2023; 381: 1350–6. 10.1126/science.adi1545 37733840

[3] Yang Y, Yang X, Liang L et al. Large-area graphene-nanomesh/carbon-nanotube hybrid membranes for ionic and molecular nanofiltration. Science 2019; 364: 1057–62. 10.1126/science.aau5321 31197007

[4] Wen Q, Jia P, Cao L et al. Electric-field-induced ionic sieving at planar graphene oxide heterojunctions for miniaturized water desalination. Adv Mater 2020; 32: 1903954. 10.1002/adma.201903954 32115802

[5] Li J, Du L, Kong X et al. Designing artificial ion channels with strict K^+^ /Na^+^ selectivity toward next-generation electric-eel-mimetic ionic power generation. Natl Sci Rev 2023; 10: nwad260. 10.1093/nsr/nwad260 37954195 PMC10632797

[6] Wang Y . Evolution of artificial channels. Natl Sci Rev 2024; 11: nwad293. 10.1093/nsr/nwad293 38116097 PMC10727835

[7] Moreno C, Vilas-Varela M, Kretz B et al. Bottom-up synthesis of multifunctional nanoporous graphene. Science 2018; 360: 199–203. 10.1126/science.aar2009 29650671

[8] Liu Y, Coppens M, Jiang Z. Mixed-dimensional membranes: chemistry and structure-property relationships. Chem Soc Rev 2021; 50: 11747–65. 10.1039/D1CS00737H 34499074

[9] Lim Y, Goh K, Wang R. The coming of age of water channels for separation membranes: from biological to biomimetic to synthetic. Chem Soc Rev 2022; 51: 4537–82. 10.1039/D1CS01061A 35575174

[10] Fang A, Kroenlein K, Riccardi D et al. Highly mechanosensitive ion channels from graphene-embedded crown ethers. Nat Mater 2019; 18: 76–81. 10.1038/s41563-018-0220-4 30478453

[11] Surwade S, Smirnov S, Vlassiouk I et al. Water desalination using nanoporous single-layer graphene. Nat Nanotechnol 2015; 10: 459–64. 10.1038/nnano.2015.37 25799521

[12] Kidambi P, Chaturvedi P, Moehring N. Subatomic species transport through atomically thin membranes: present and future applications. Science 2021; 374: eabd7687. 10.1126/science.abd7687 34735245

[13] Wang S, Yang L, He G et al. Two-dimensional nanochannel membranes for molecular and ionic separations. Chem Soc Rev 2020; 49: 1071–89. 10.1039/C9CS00751B 31971530

[14] Noy A, Wanunu M. A new type of artificial water channels. Nat Nanotechnol 2020; 15: 9–10. 10.1038/s41565-019-0617-5 31853008

[15] Li Z, Gadipelli S, Li H et al. Tuning the interlayer spacing of graphene laminate films for efficient pore utilization towards compact capacitive energy storage. Nat Energy 2020; 5: 160–8. 10.1038/s41560-020-0560-6

[16] Cheng C, Jiang G, Simon G et al. Low-voltage electrostatic modulation of ion diffusion through layered graphene-based nanoporous membranes. Nat Nanotechnol 2018; 13: 685–90. 10.1038/s41565-018-0181-4 29967459

[17] Wang Z, Ma C, Xu C et al. Graphene oxide nanofiltration membranes for desalination under realistic conditions. Nat Sustain 2021; 4: 402–8. 10.1038/s41893-020-00674-3

[18] Ries L, Petit E, Michel T et al. Enhanced sieving from exfoliated MoS_2_ membranes via covalent functionalization. Nat Mater 2019; 18: 1112–7. 10.1038/s41563-019-0464-7 31451779

[19] Shen J, Liu G, Han Y et al. Artificial channels for confined mass transport at the sub-nanometre scale. Nat Rev Mater 2021; 6: 294–312. 10.1038/s41578-020-00268-7

[20] Shih C, Vijayaraghavan A, Krishnan R et al. Bi- and trilayer graphene solutions. Nat Nanotechnol 2011; 6: 439–45. 10.1038/nnano.2011.94 21706026

[21] Xu M, Liang T, Shi M et al. Graphene-like two-dimensional materials. Chem Rev 2013; 113: 3766–98. 10.1021/cr300263a 23286380

[22] Ding L, Li L, Liu Y et al. Effective ion sieving with Ti_3_C_2_T_x_ MXene membranes for production of drinking water from seawater. Nat Sustain 2020; 3: 296–302. 10.1038/s41893-020-0474-0

[23] Park H, Kamcev J, Robeson L et al. Maximizing the right stuff: the trade-off between membrane permeability and selectivity. Science 2017; 356: eaab0530. 10.1126/science.aab0530 28619885

[24] Wang P, Wang M, Liu F et al. Ultrafast ion sieving using nanoporous polymeric membranes. Nat Commun 2018; 9: 569. 10.1038/s41467-018-02941-6 29422511 PMC5805712

[25] Lai H, Benedetti F, Ahn J et al. Hydrocarbon ladder polymers with ultrahigh permselectivity for membrane gas separations. Science 2022; 375: 1390–2. 10.1126/science.abl7163 35324307

[26] Zuo P, Ye C, Jiao Z et al. Near-frictionless ion transport within triazine framework membranes. Nature 2023; 617: 299–305. 10.1038/s41586-023-05888-x 37100908 PMC10131500

[27] Epsztein R, DuChanois R, Ritt C et al. Towards single-species selectivity of membranes with subnanometre pores. Nat Nanotechnol 2020; 15: 426–36. 10.1038/s41565-020-0713-6 32533116

[28] Mi B . Scaling up nanoporous graphene membranesc. Science 2019; 364: 1033–4. 10.1126/science.aax3103 31197000

[29] Liu G, Jin W, Xu N. Graphene-based membranes. Chem Soc Rev 2015; 44: 5016–30. 10.1039/C4CS00423J 25980986

[30] Prozorovska L, Kidambi P. State-of-the-art and future prospects for atomically thin membranes from 2D materials. Adv Mater 2018; 30: 1801179. 10.1002/adma.201801179 30085371

[31] Tan C, Cao X, Wu X et al. Recent advances in ultrathin two-dimensional nanomaterials. Chem Rev 2017; 117: 6225–331. 10.1021/acs.chemrev.6b00558 28306244

[32] Cohen-Tanugi D, Grossman J. Water desalination across nanoporous graphene. Nano Lett 2012; 12: 3602–8. 10.1021/nl3012853 22668008

[33] Zhang H, Li X, Hou J et al. Angstrom-scale ion channels towards single-ion selectivity. Chem Soc Rev 2022; 51: 2224–54. 10.1039/D1CS00582K 35225300

[34] Tunuguntla R, Henley R, Yao Y et al. Enhanced water permeability and tunable ion selectivity in subnanometer carbon nanotube porins. Science 2017; 357: 792–6. 10.1126/science.aan2438 28839070

[35] Heiranian M, Farimani A, Aluru N. Water desalination with a single-layer MoS_2_ nanopore. Nat Commun 2015; 6: 8616. 10.1038/ncomms9616 26465062 PMC4634321

[36] Ritt C, Werber J, Deshmukh A et al. Monte Carlo simulations of framework defects in layered two dimensional nanomaterial desalination membranes: implications for permeability and selectivity. Environ Sci Technol 2019; 53: 6214–24. 10.1021/acs.est.8b06880 31066551

[37] Abraham J, Vasu K, Williams C et al. Tunable sieving of ions using graphene oxide membranes. Nat Nanotechnol 2017; 12: 546–50. 10.1038/nnano.2017.21 28369049

[38] Zheng S, Tu Q, Urban J et al. Swelling of graphene oxide membranes in aqueous solution: characterization of interlayer spacing and insight into water transport mechanisms. ACS Nano 2017; 11: 6440–50. 10.1021/acsnano.7b02999 28570812

[39] Mi B . Graphene oxide membranes for ionic and molecular sieving. Science 2014; 343: 740–2. 10.1126/science.1250247 24531961

[40] Wan S, Chen Y, Fang S et al. High-strength scalable graphene sheets by freezing stretch-induced alignment. Nat Mater 2021; 20: 624–31. 10.1038/s41563-020-00892-2 33542472

[41] Gopinadhan K, Hu S, Esfandiar A et al. Complete steric exclusion of ions and proton transport through confined monolayer water. Science 2019; 363: 145–7. 10.1126/science.aau6771 30630924

[42] Wang L, Boutilier M, Kidambi P et al. Fundamental transport mechanisms, fabrication and potential applications of nanoporous atomically thin membranes. Nat Nanotechnol 2017; 12: 509–22. 10.1038/nnano.2017.72 28584292

[43] Fang H, Ni K, Wu J et al. The effects of hydrogen bonding on the shear viscosity of liquid water. Int J Sediment Res 2019; 34: 8–13. 10.1016/j.ijsrc.2018.10.008

[44] Itoh Y, Chen S, Hirahara R et al. Ultrafast water permeation through nanochannels with a densely fluorous interior surface. Science 2022; 376: 738–43. 10.1126/science.abd0966 35549437

[45] Yuan Z, He G, Faucher S et al. Direct chemical vapor deposition synthesis of porous single-layer graphene membranes with high gas permeances and selectivities. Adv Mater 2021; 33: 2104308. 10.1002/adma.202104308 34510595

[46] Celebi K, Buchheim J, Wyss R et al. Ultimate permeation across atomically thin porous graphene. Science 2014; 344: 289–92. 10.1126/science.1249097 24744372

[47] Huang G, Ghalei B, Pournaghshband Isfahani A et al. Overcoming humidity-induced swelling of graphene oxide-based hydrogen membranes using charge-compensating nanodiamonds. Nat Energy 2021; 6: 1176–87. 10.1038/s41560-021-00946-y

[48] Zhang W, Yin M, Zhao Q et al. Graphene oxide membranes with stable porous structure for ultrafast water transport. Nat Nanotechnol 2021; 16: 337–43. 10.1038/s41565-020-00833-9 33479540

[49] Kang J, Ko Y, Kim J et al. Microwave-assisted design of nanoporous graphene membrane for ultrafast and switchable organic solvent nanofiltration. Nat Commun 2023; 14: 901. 10.1038/s41467-023-36524-x 36797272 PMC9935848

[50] Al-Kharabsheh S . An innovative reverse osmosis desalination system using hydrostatic pressure. Desalination 2006; 196: 210–4. 10.1016/j.desal.2005.11.023

[51] Jain T, Rasera B, Guerrero R et al. Heterogeneous sub-continuum ionic transport in statistically isolated graphene nanopores. Nat Nanotechnol 2015; 10: 1053–7. 10.1038/nnano.2015.222 26436566

[52] Thomas M, Corry B, Hilder T. What have we learnt about the mechanisms of rapid water transport, ion rejection and selectivity in nanopores from molecular simulation? Small 2014; 10: 1453–65. 10.1002/smll.201302968 24851242

[53] Williams C, Carbone P. Selective removal of technetium from water using graphene oxide membranes. Environ Sci Technol 2016; 50: 3875–81. 10.1021/acs.est.5b06032 26954102

[54] Song C, Corry B. Intrinsic ion selectivity of narrow hydrophobic pores. J Phys Chem B 2009; 113: 7642–9. 10.1021/jp810102u 19419185

[55] Gong X, Li J, Lu H et al. A charge-driven molecular water pump. Nat Nanotechnol 2007; 2: 709–12. 10.1038/nnano.2007.320 18654410

[56] Wu X, Cui X, Wang Q et al. Manipulating the cross-layer channels in g-C_3_N_4_ nanosheet membranes for enhanced molecular transport. J Mater Chem A 2021; 9: 4193–202. 10.1039/D0TA10236A

