# Supplementary material for: Fast water transport and ionic sieving in ultrathin stacked nanoporous 2D membranes
Source: Natl Sci Rev. 2025 Jan 6;12(3):nwae482. doi: 10.1093/nsr/nwae482 (PMC11809252; doi:10.1093/nsr/nwae482)
Supplement: nwae482_Supplemental_Files [file nwae482_supplemental_files.zip › Supplementary data.pdf]

Supplementary information

Fast water transport and ionic sieving in ultrathin stacked nanoporous two-dimensional membranes

Jingfeng Wang[1,†], Xiaoming Zhang[1,†], Zehua Yu[1,†], Yuyan Gao[3], Qingqing Lu[1], Chao Ma[2], Kang Liu[1,*], Quan Yuan[1,2,*], Yanbing Yang[1,*]

[1] College of Chemistry and Molecular Sciences, Key Laboratory of Biomedical Polymers of Ministry of Education, School of Power and Mechanical Engineering, Institute of Molecular Medicine, Renmin Hospital of Wuhan University, School of Microelectronics, Wuhan University, Wuhan 430072, China

[2] Molecular Science and Biomedicine Laboratory (MBL), State Key Laboratory of Chemo/Biosensing and Chemometrics, College of Chemistry and Chemical Engineering, College of Materials Science and Engineering, Hunan University, Changsha 410082, China

[3] Department of Engineering Science and Mechanics, The Pennsylvania State University, University Park, PA 16802, USA

*Corresponding Authors: yangyanbing@whu.edu.cn; yuanquan@whu.edu.cn; kang.liu@whu.edu.cn

[†]Equally contributed to this work.

This file includes:

Materials and Methods

Figures S1 to S44

Table S1

Caption of Video S1

References

Other Supplementary Material for this manuscript includes the following:

Video S1

Table of contents

Materials and Methods........ *5*

Material characterization........ 5

Preparation of monolayer graphene........ 5

Fabrication of stacked nanoporous graphene membranes........ 5

Mechanical property measurements........ 6

Water transport and ionic/molecular rejection with the RO cross-flow system........ 6

Membrane biofouling tests........ 7

Nanoindentation of the membrane using molecular dynamic simulation........ 8

Exploration of molecule/ion transport behavior using MD simulation........ 10

Supplementary Figures........ *11*

Figure S1. Schematic illustration of the process used to fabricate the SNGMs........ 11

Figure S2. Aberration-corrected STEM image of the pristine monolayer graphene........ 11

Figure S3. Pore size distributions of the SNGMs prepared by O_2 plasma etching time of 10 s........ 12

Figure S4. Aberration-corrected STEM image and pore size distributions of the SNGMs prepared by different O_2 plasma etching time of 5 s and 20 s, respectively........ 12

Figure S5. Raman spectra of monolayer graphene nanomesh, SNGMs assembled by bilayer and trilayer graphene nanomesh........ 13

Figure S6. XPS C1s core of the SNGMs........ 13

Figure S7. SEM micrograph of the silicon nitride substrate covered by SNGMs........ 14

Figure S8. Layouts of SNGMs........ 14

Figure S9. Schematic illustration of the SNGMs in MD simulations........ 15

Figure S10. Indentation force-displacement curves of monolayer graphene nanomesh, and SNGMs assembled by bilayer and trilayer graphene nanomesh in MD simulations........ 15

Figure S11. Different indentation depth at 100 nN force on the monolayer graphene nanomesh, SNGMs assembled by bilayer and trilayer graphene nanomesh in MD simulations........ 16

Figure S12. The spatial distribution of the SNGMs at 100 nN force in MD simulations........ 16

Figure S13. Young's modulus of monolayer graphene nanomesh, and SNGMs assembled by bilayer and trilayer graphene nanomesh in MD simulations........ 17

Figure S14. A snapshot of the water transport in the monolayer graphene nanomesh........ 17

Figure S15. A snapshot of the water transport in the SNGM assembled by bilayer graphene nanomesh........ 18

Figure S16. A snapshot of water molecules transport processes in the in-plane nanopores and 2D nanochannels of the SNGMs........ 18

Figure S17. Hydroxy bonds distribution among water molecules in the monolayer graphene nanomesh........ 19

Figure S18. Hydroxy bonds distribution between water molecules and the monolayer graphene nanomesh........ 19

Figure S19. Hydroxy bonds distribution among water molecules in the SNGM assembled by bilayer

graphene nanomesh. 20
Figure S20. Hydroxy bonds distribution between water molecules and the SNGM assembled by bilayer graphene nanomesh. 20
Figure S21. Hydroxy bonds distribution between water molecules and the SNGM assembled by trilayer graphene nanomesh.. 21
Figure S22. Contact angle of the SNGMs. 21
Figure S23. Time-averaged water molecules transport velocity in the monolayer graphene nanomesh, SNGMs assembled by bilayer and trilayer graphene nanomesh (x-y plane).................. 22
Figure S24. Effect of pore offset of membrane configurational parameters on the water flowrate in the SNGM assembled by bilayer graphene nanomesh. 22
Figure S25. Effect of pore offset of membrane configurational parameters on the water flowrate in the SNGM assembled by trilayer graphene nanomesh... 23
Figure S26. Schematic illustration of the RO cross-flow filtration apparatus................................. 23
Figure S27. Photograph of the membrane cell in the RO cross-flow filtration apparatus................ 24
Figure S28. Photograph of the SNGM assembled by trilayer graphene nanomesh supported by SWNT suspended on a PET substrate. 24
Figure S29. SEM image of the SNGM assembled by trilayer graphene nanomesh supported by SWNT.. 25
Figure S30. TEM image of the SNGM assembled by trilayer graphene nanomesh supported by SWNT.. 25
Figure S31. AFM image and height profile of the SNGM assembled by trilayer graphene nanomesh supported by SWNT. 26
Figure S32. Modulus map and modulus distribution of SNGM assembled by trilayer graphene nanomesh supported by SWNT. 26
Figure S33. Water permeation performance of the SNGMs.. 27
Figure S34. Normalized absorption spectra of the dye molecules (Rhodamine B, RhB) and antibiotics (tetracycline, TC) before and after filtration.. 27
Figure S35. Water permeability of monolayer graphene nanomesh, and SNGMs assembled by bilayer and trilayer graphene nanomesh for 2000 ppm NaCl solution.. 28
Figure S36. Reverse osmosis separation performance. 28
Figure S37. Water permeability and salt rejection of the SNGM assembled by trilayer graphene nanomesh with O_2 plasma-etching time of 5, 10, and 20 s, respectively... 29
Figure S38. Analysis of the adsorption percentage of SNGM assembled by trilayer graphene nanomesh for salt, dye molecules and antibiotic during the filtration tests..................................... 29
Figure S39. The calculated water flowrate in NaCl solutions and NaCl rejection of the SNGMs with the pore size of 8 Å in MD simulations.. 30
Figure S40. Salt rejection of the SNGM assembled by trilayer graphene nanomesh measured at pH values of 3.0 and 7.0 for 2000 ppm NaCl solution.. 30
Figure S41. Long-term stability of the monolayer graphene nanomesh.. 31
Figure S42. Long-term stability of the SNGM assembled by bilayer graphene nanomesh. 31
Figure S43. Cycle performance of the SNGM assembled by trilayer graphene nanomesh. 32
Figure S44. Fluorescence microscopy images of *S. putrefaciens* cells on the SNGM assembled by trilayer graphene nanomesh and CTA membrane after 24 h of operation. 32

Supplementary Table *33*

Table S1. XPS survey for CVDG, monolayer graphene nanomesh, SNGMs assembled by bilayer and trilayer graphene nanomesh. 33

Table S2. Comparison of the water permeability and salt rejection performance of the SNGM with state-of-the-art membranes reported. 34

Supplementary Video *36*

Video S1. Water molecules transport processes in the in-plane nanopores and 2D nanochannels of the SNGMs. 36

References *37*

Materials and Methods

Material characterization. Scanning electron microscope (SEM) images were taken with Zeiss Merlin Compact (FEI Verios 460). Transmission electron microscopy (TEM) images were obtained using a JEM-2100 Plus operated at 200 kV. Aberration-corrected scanning transmission electron microscopy (STEM) images were collected on a JEM-ARM200CF operated at 80 kV. The cross-section TEM images of SNGMs were obtained by a focused ion beam (FIB) method (FEI Strata 400S). X-ray diffraction (XRD) measurements were performed using a Rigaku Miniflex600 detector with a Cu Kα radiation (λ = 1.5410 Å). Atomic force microscopy (AFM) was operated using Park nx10. The dynamic contact angles of the membranes were measured using the KRUSS DSA100S, Germany. X-ray photoelectron spectroscopy (XPS) measurements were performed using a Thermo Fisher Scientific ESCALAB250Xi XPS spectrometer. The surface properties of the membranes were determined by FTIR (FTIR5700), and Raman spectra (HORIBA Jobin Yvon XploRA Plus). The absorption spectra were recorded on a UV 2550 spectrophotometer (Shimadzu). Fluorescence microscopy images of the *S. putrefaciens* cells were collected on an Olympus FV1200 Confocal Microscopy Imaging System.

Preparation of monolayer graphene. The monolayer graphene was synthesized using ambient-pressure chemical vapor deposition (CVD) on a copper foil catalyst in a methane and hydrogen atmosphere. Before synthesis, the copper foil in the tubular furnace with quartz tube was annealed in a constant hydrogen atmosphere flow of 7 sccm at the temperature of 1050 ^{o}C for 30 min. After the 30 min growth in a constant methane atmosphere flow of 10 sccm, the furnace was cooled to room temperature under a hydrogen atmosphere and the monolayer graphene was obtained.

Fabrication of stacked nanoporous graphene membranes. The stacked nanoporous graphene membranes (SNGMs) were assembled by different layers of monolayer graphene nanomesh. The monolayer graphene nanomesh were obtained by using the meso-SiO_2 as the porous template to introduce the nanopores with uniform pore size into the monolayer graphene. Specifically, the meso-SiO_2 precursor solution including 0.08 g cetyltrimethyl ammonium bromide, 15 mL ethanol, 35 mL water, 5 μL concentrated ammonia aqueous solution, and 40 μL tetraethyl orthosilicate, was used to grow meso-SiO_2 porous template. The growth temperature was set at 60 ^{o}C and growth time is 4 h. After rinsing with water, the meso-SiO_2 template coated graphene membranes were dried in the atmosphere and aged

overnight at 100 ^{o}C. The ethanol solution containing 0.1 M hydrochloric acid was used to remove the surfactant, and then the membranes were washed with water to remove ethanol. The nanopores in the graphene nanomesh were introduced by an O_2 plasma process (45 W, 20 mtorr), and the pore size and pore density were controlled through regulating the O_2 plasma treatment time from 5 s to 20 s. After immersing in HF vapor to remove the meso-SiO_2 porous template, a monolayer graphene nanomesh was obtained. The SNGMs assembled by bilayer and trilayer graphene nanomesh were fabricated by sequentially stacking the monolayer graphene nanomesh with the assistance of poly(methyl methacrylate) (PMMA). Monolayer graphene nanomesh, SNGMs assembled by bilayer, and trilayer graphene nanomesh were prepared by O_2 plasma etching time of 10 s for water transport and ionic/molecular rejection measurement.

Mechanical property measurements. The samples were prepared by transferring the as-prepared SNGMs onto a silicon nitride substrate. The nanoindentation tests were conducted in ambient conditions with an AFM (Park nx10 systems). Before the indentation process, the samples were scanned for 2 h to minimize the thermal drift of the piezo actuators, and the indentation position was found accurately by scanning each graphene nanomesh membrane in non-contact mode. The loading/unloading curves were collected in force/displacement spectroscopy mode for each graphene nanomesh membrane to multiple depths. Modulus measurements were carried out at room temperature and relative humidity of 20%. The modulus map was obtained over an area of $2.5 \times 2.5\ \mu m^2$ SNGM assembled by trilayer graphene nanomesh supported by a single-walled carbon nanotube (SWNT).

Water transport and ionic/molecular rejection with the RO cross-flow system. All the water permeability and molecular/ionic sieving measurements were measured with a homemade cross-flow RO apparatus. The as-prepared SNGMs were mechanically supported by SWNT membrane (~50 to 500 nm pore size) and sealed on a drilled PET film to produce an effective membrane area of 0.13 cm^2 for transport. The measurements were performed at room temperature with a cross-flow velocity of 0.4 cm s^{-1}. All the separation performance including water permeability and ion rejection were collected once the fabricated membranes reached a steady state at 0.15 bar for at least 30 min. The mass change of the permeate side was recorded at specific time intervals with a Mettler Toledo AL204 balance after the system was stabilized (~30 minutes). The conductivity changes of the permeate solution were recorded by a Mettler Toledo FE38 conductivity meter to assess the salt rejection performance

of the membranes. Organic dye including Rhodamine B (RhB) (dispersed in water) with a concentration of 20 ppm and tetracycline (TC) antibiotic molecule (dispersed in water) with a concentration of 200 ppm were tested separately. The absorption curves of the solution in the permeate side RhB (554 nm) and TC (275 nm, 355 nm) were recorded using a UV-vis spectrometer to quantify the rejection ratio [1,2]. The rejection performance of the SNGM towards boron was tested with a solution of 5 ppm boron at pH 7. The B concentration was measured using Inductively Coupled Plasma-Optical Emission Spectroscopy (ICP-OES) (PlasmaQuant, PQ9000). All the filtration operations were performed with at least three membranes.

The rejection rate was calculated according to equation (1):

$$\mathrm{R} = 1 - \frac{C_\mathrm{p}}{C_\mathrm{f}} \tag{1}$$

where C_p and C_f are the concentrations of salt or molecule in the permeate side and feed side, respectively.

The water permeability (J_w) was calculated according to equation (2):

$$J_\mathrm{w} = \Delta w/st \tag{2}$$

where Δw is the mass change of the feed solution, s is the effective membrane area and t is the recording time.

The percentage of adsorption Ads. (%) of the SNGMs for the solute molecules in the system can be calculated by the following relation (3):

$$\text{Ads. (\%)} = \frac{V_\mathrm{f}C_\mathrm{f} - (V_\mathrm{r}C_\mathrm{r} + V_\mathrm{p}C_\mathrm{p})}{V_\mathrm{f}C_\mathrm{f}} \times 100\ (\%) \tag{3}$$

where C_f, C_r, and C_p are the concentrations of solute molecules in the feed, retentate and permeate side, respectively [3,4].

Membrane biofouling tests. *S. putrefaciens* was used for the biofouling tests. The bacteria were cultured at 30 $^\circ$C in Luria-Bertani (LB) medium, and then collected by centrifugation and suspended in PB buffer. The bacteria were stained with Calcein-AM. The biofouling experiment was carried out by injecting the bacteria solution into the feed solution. The cross-flow RO apparatus was operated for 24 h and the SNGM assembled by trilayer graphene nanomesh and CTA membrane were then disassembled from the apparatus for the fluorescent microscopy observation.

Nanoindentation of the membrane using molecular dynamic simulation. The objective of the simulation section is to evaluate the enhancement in mechanical properties, particularly the total resistance force, of SNGMs in comparison to single-layer porous graphene. To accomplish this, we employed the open-source molecular dynamics software, Large-scale Atomic/Molecular Massively Parallel Simulator (LAMMPS), to simulate nanoindentation on graphene nanomesh membranes with varying layers, ranging from single-layer to double and triple layers [5]. This approach enabled us to investigate the effect of layer numbers on the mechanical response of graphene nanomesh.

Firstly, the graphene nanomesh membrane was designed with a customized MATLAB code. The dimension of the graphene membrane is 42 nm × 42 nm, with a fixed interatomic distance of 0.142 nm between carbon atoms [6]. According to experimental data, we set that the holes on the membrane have a diameter of 1 nm and the overall porosity is controlled at 10%. We assumed a uniform distribution of holes on the membrane and utilized a square-shaped layout. Figure S8 illustrates the arrangement of the multilayer membranes, with the inter-hole distance adjusted to maintain the 10% porosity. To gain a deeper understanding of how the porous structure affects the overall properties of a sheet, we assumed that there is no overlap of holes between each layer. In the case of a triple-layer graphene sheet, the hole layout of layer 1 (the bottom layer) and layer 3 (the top layer) is identical, while layer 2 (the middle layer) has a complementary pattern of holes. Similarly, for single-layer graphene nanomesh, the hole layout follows that of layer 1, while for double-layer porous graphene, the hole layout is a combination of layer 1 and layer 2. We have set the distance between each layer to 0.34 nm [7].

Next, we employed the LAMMPS to simulate the nanoindentation process (Figure S9). To describe the atomic interactions among carbon atoms, we utilized the adaptive intermolecular reactive empirical bond order potential (AIREBO) [8], which is a widely selected potential for graphene [7,9]. The AIREBO potential is composed of three terms:

$$E = \frac{1}{2}\sum_{i}\sum_{j\neq i}\left[E_{ij}^{REBO}+E_{ij}^{LJ}+\sum_{k\neq i,j}\sum_{l\neq i,j,k}E_{kijl}^{TORSION}\right] \tag{4}$$

where the E_{ij}^{REBO} term describes the short-ranged carbon-carbon reactions; E_{ij}^{LJ} is the Lennard-Jones potential and takes the interlayer interactions into account; $E_{kijl}^{TORSION}$ describes various dihedral angle preferences.

In the simulation, we set the time step in the LAMMPS code to be 1 fs, and the graphene membrane was equilibrated for a total of 5 ps under the Nose-Hoover thermostat (NVT) at 300 K. This allowed the system to reach a stable state before proceeding with the nanoindentation simulation. Then a spherical and rigid indenter with a diameter of 10 nm moved toward the graphene sheet and hit the center of the sheet. The simulation was carried out using the NVT setting at a temperature of 300 K. The boundaries of the graphene sheet were clamped (blue area in Figure S9), and the atoms in the interjacent circular area (red area in Figure S9) with a diameter of 39 nm were allowed to deform when the indenter contact the sheet. The interactive force between the indenter and each atom in the graphene sheet was determined using the following equation:

$$F(r) = -K(r-R)^2 \tag{5}$$

where r is the distance between each atom and the center of the indenter, R is the radius of the indenter, and K is a force constant. In this study, K was set to 10 eV/Å^3 based on previously published literature [7]. The loading speed of the indenter was set to 0.25 Å/ps, and the total reaction force from the graphene sheet was recorded over time. The force-indentation depth curve (Figure S10) obtained from the simulation showed an increase in force with an increasing number of graphene layers. This increase in force indicated an enhancement of resistance to external loads. The relationship between force and indentation depth was estimated numerically using the following equation [9]:

$$F = \sigma_0^{2D}(\pi a)(\delta/a) + E^{2D}(q^3 a)(\delta/a)^3 \tag{6}$$

where F is the force applied onto the graphene membrane, σ_0^{2D} is the in-plane pretension stress of the graphene, δ is the indentation depth, a is the diameter of the graphene membrane, E^{2D} is the in-plane elastic modulus and can be transferred into the bulk modulus E by E^{2D}/t, where t is the total thickness of the graphene sheet. q is a constant that relates with Poisson's ratio v : $q = 1/\left(1.05\text{-}0.15v\text{-}0.16v^2\right)$. Based on the results of the fitting curve, the elastic modulus (or Young's modulus) for single, double, and triple-layer porous graphene sheets was found to be 0.815 TPa, 0.949 TPa, and 0.894 TPa, respectively (Figure S13). The elastic modulus exhibits a value below 1 TPa owing to the existence of 10% porosity. These findings indicate that the mechanical strength remains at the same level, and total resistance force can be enhanced by the stacking of multilayer graphene sheets.

Exploration of molecule/ion transport behavior using MD simulation. MD simulations were carried out to investigate the desalination process of SNGMs using the LAMMPS package. The simulation cell is composed of feed-side water solution and permeate-side water solution separated by the SNGMs. A rigid piston was used to apply a transmembrane pressure to the system to observe the water molecules transport processes. To obtain accurate statistics, more than 3 independent simulations were conducted for each set of parameters. The SNGMs were sandwiched by a saline chamber on the left and a pure water chamber on the right. A 20000 ppm NaCl solution was chosen to imitate the saline solution. Two rigid impermeable graphene plates were placed on the ends of the saline and water chamber to control the liquid pressure. The pressures on the saline and water sides were set as P_s = 201 MPa and P_w = 1 MPa, respectively, resulting in a pressure difference ΔP of 200 MPa. In all simulations, we assumed the membranes is rigid because the deformation effect of SNGMs on desalination performance was found to be marginal under high hydraulic pressure. The TIP4P-Ew model for water molecules and the corresponding parameters for ions proposed by Joung et al. were used [10]. The LJ parameters for carbon atoms in graphene were obtained from the work of Beu et al [11]. For the hydrogenated groups, the LJ parameters and atomic charges used were from the study of Mooney et al [12]. The Lorentz-Berthelot mixing rules were used to calculate all pairwise Lennard-Jones terms. The simulations were carried out in the NVT ensemble at a temperature of 300 K, utilizing a Nose-Hoover thermostat with a damping factor of 100.0.

The number of water molecules and ions in the feed, interlayer of SNGMs, and permeate regions over time were recorded in each simulation. The number of feed waters decreases at a constant rate, while the number of permeate waters increases at the same rate, despite the number of water molecules in the interlayer remaining constant.

Supplementary Figures

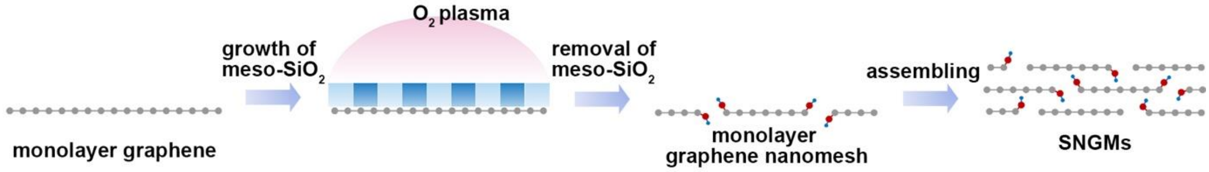

Figure S1. Schematic illustration of the process used to fabricate the SNGMs.

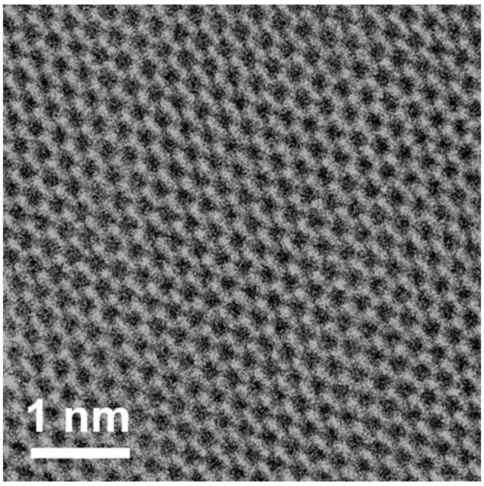

Figure S2. Aberration-corrected STEM image of the pristine monolayer graphene.

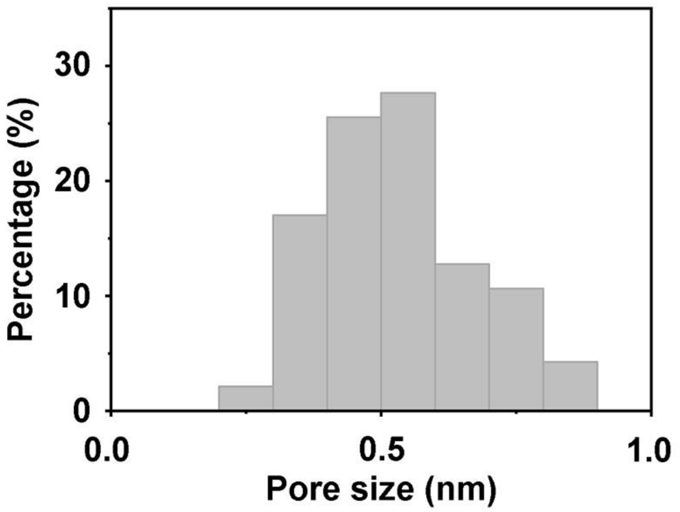

Figure S3. Pore size distributions of the SNGMs prepared by O_2 plasma etching time of 10 s.

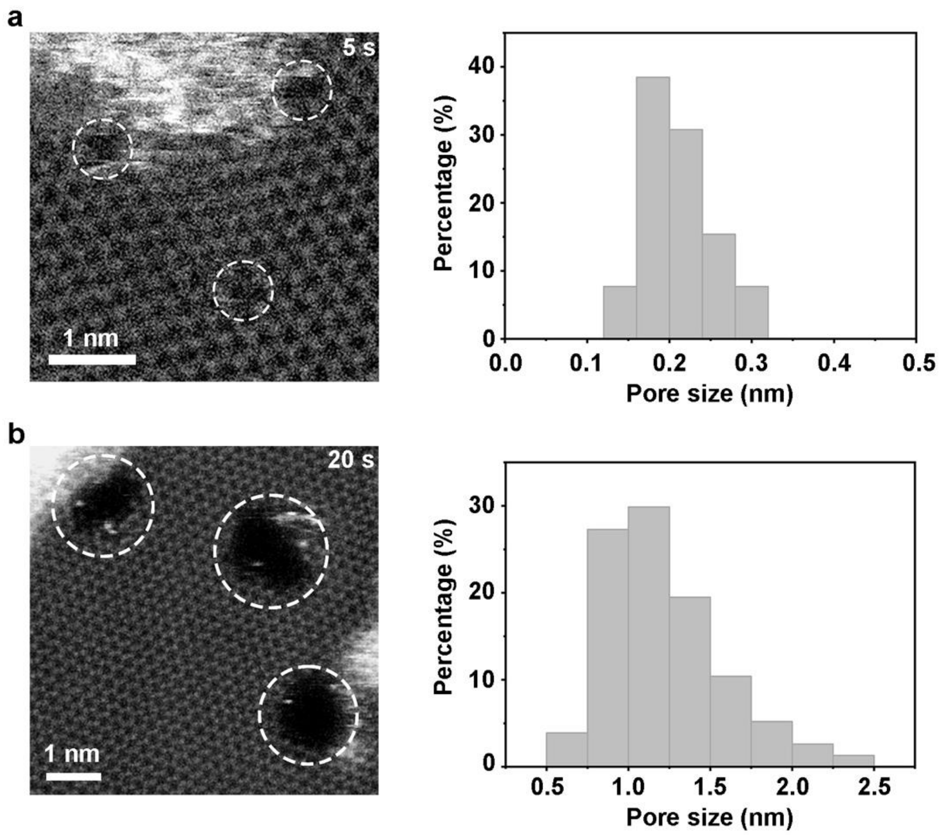

Figure S4. Aberration-corrected STEM image and pore size distributions of the SNGMs prepared by different O_2 plasma etching time of 5 s and 20 s, respectively.

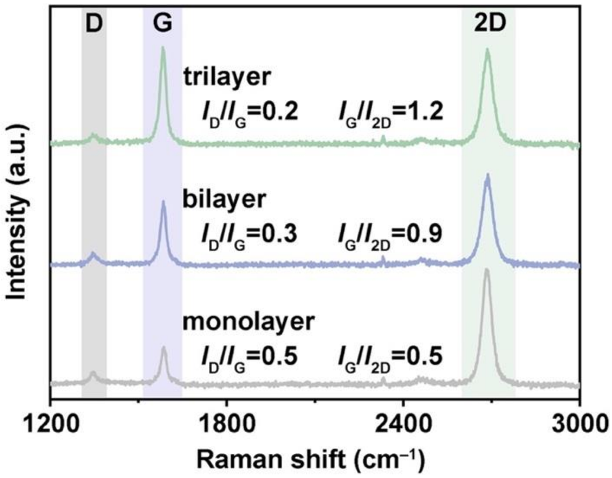

Figure S5. Raman spectra of monolayer graphene nanomesh, SNGMs assembled by bilayer and trilayer graphene nanomesh.

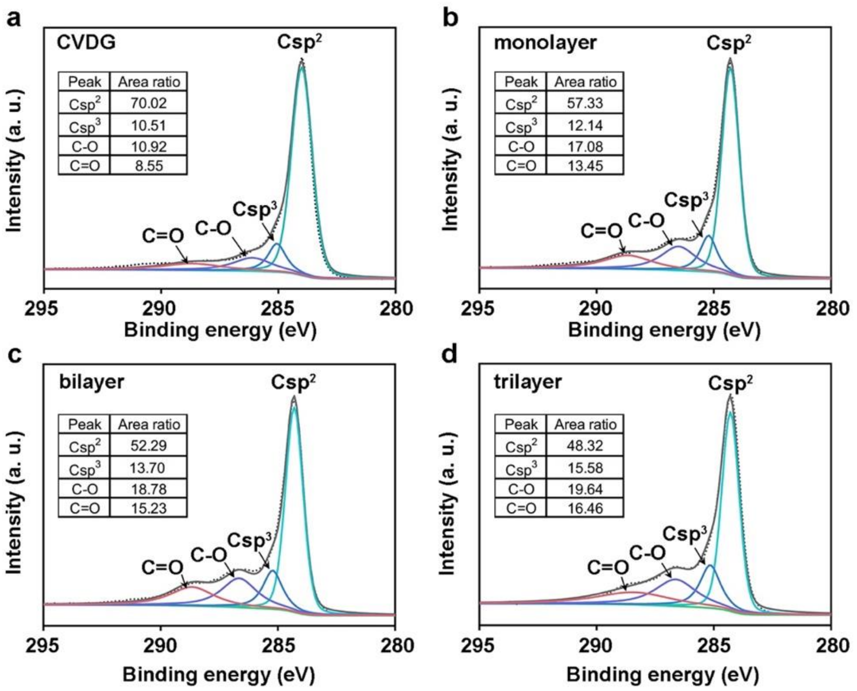

Figure S6. XPS C1s core of the SNGMs. (a) CVDG. (b) monolayer graphene nanomesh. (c) SNGMs assembled by bilayer graphene nanomesh and (d) SNGMs assembled by trilayer graphene nanomesh. Insert of (a), (b), (c), and (d): Corresponding area ratio of Csp^2, Csp^3, C-O, and C=O, respectively.

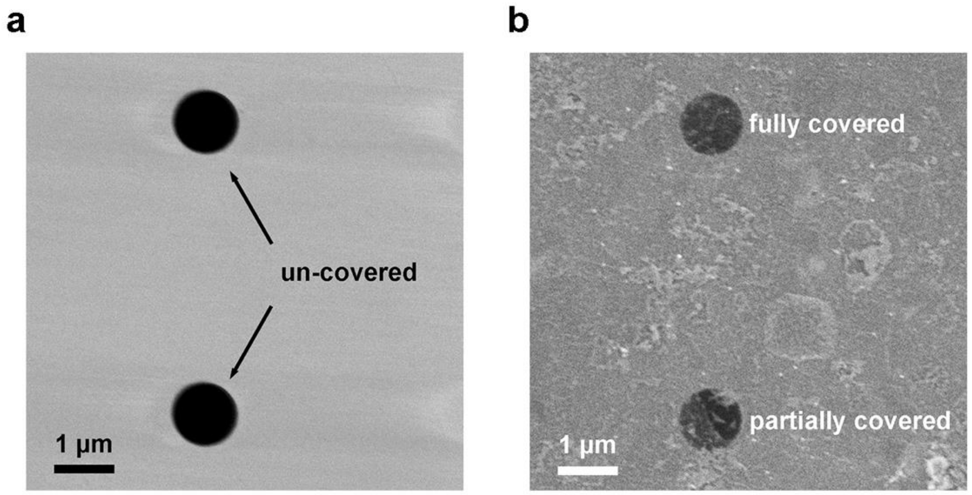

Figure S7. SEM micrograph of the silicon nitride substrate covered by SNGMs. **a**, un-covered holes; **b**, a hole fully covered by the SNGMs, and a hole partially covered by the SNGMs.

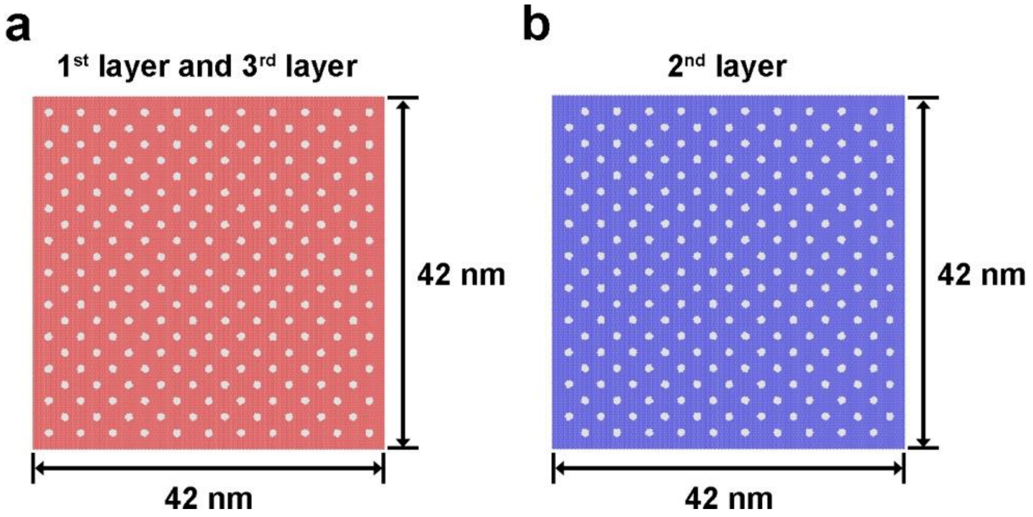

Figure S8. Layouts of SNGMs. (a) 1st & 3rd layer layout; (b) 2nd layer layout.

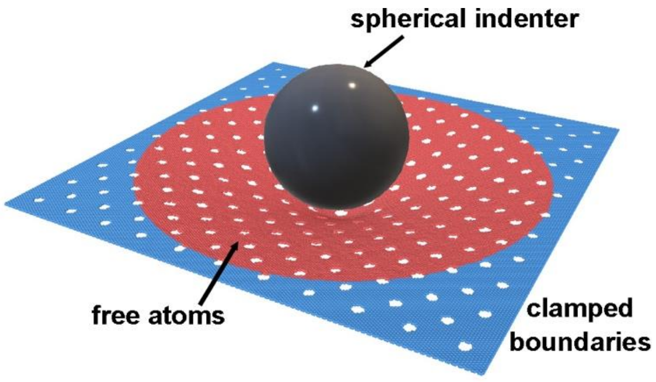

Figure S9. Schematic illustration of the SNGMs in MD simulations.

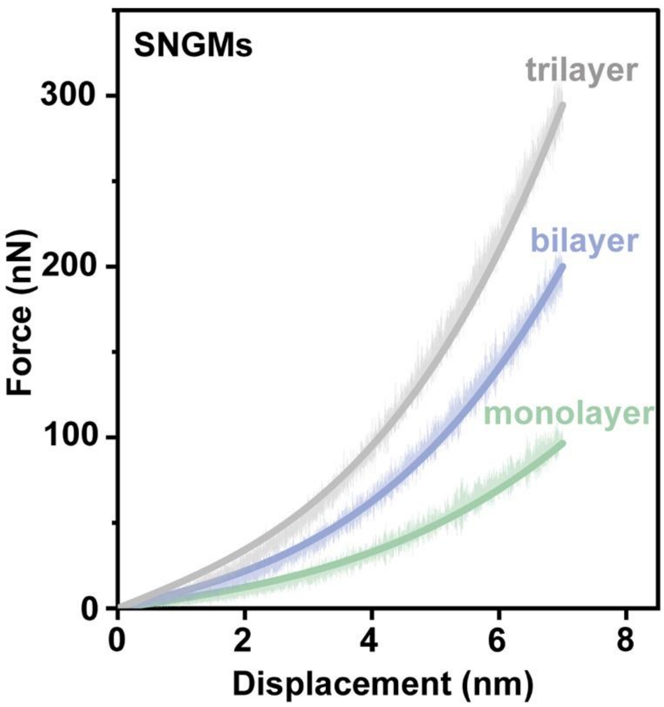

Figure S10. Indentation force-displacement curves of monolayer graphene nanomesh, and SNGMs assembled by bilayer and trilayer graphene nanomesh in MD simulations.

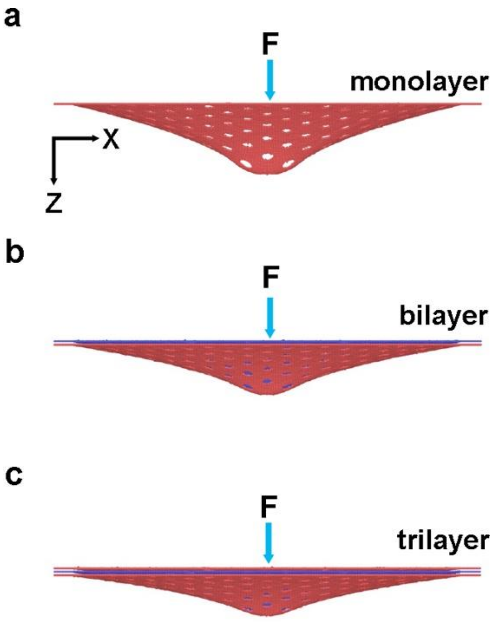

Figure S11. Different indentation depth at 100 nN force on the monolayer graphene nanomesh, SNGMs assembled by bilayer and trilayer graphene nanomesh in MD simulations.

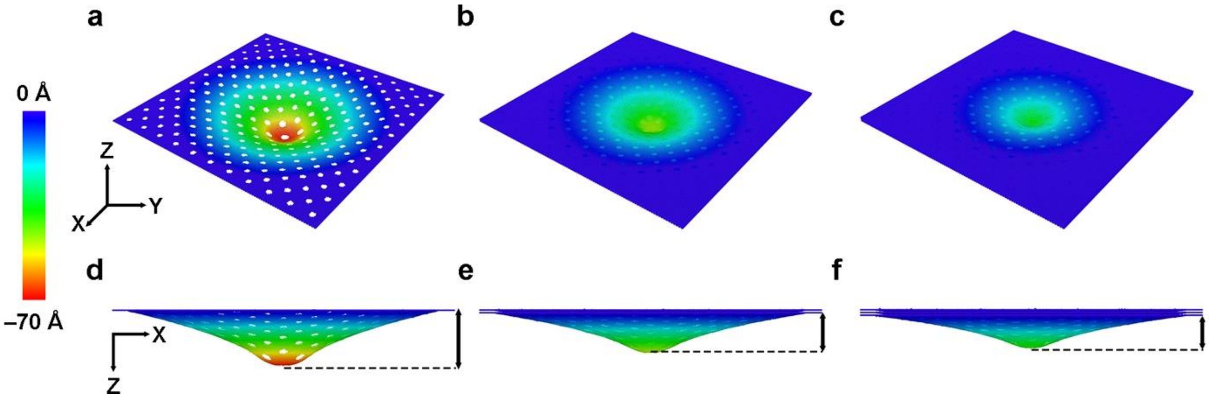

Figure S12. The spatial distribution of the SNGMs at 100 nN force in MD simulations. The indentation depth of the monolayer graphene nanomesh is 70 Å in (a, d); the SNGMs assembled by bilayer graphene nanomesh is 51 Å in (b, e) and trilayer graphene nanomesh is 42 Å in (c, f). The top and bottom figures correspond to XYZ and XZ views, respectively.

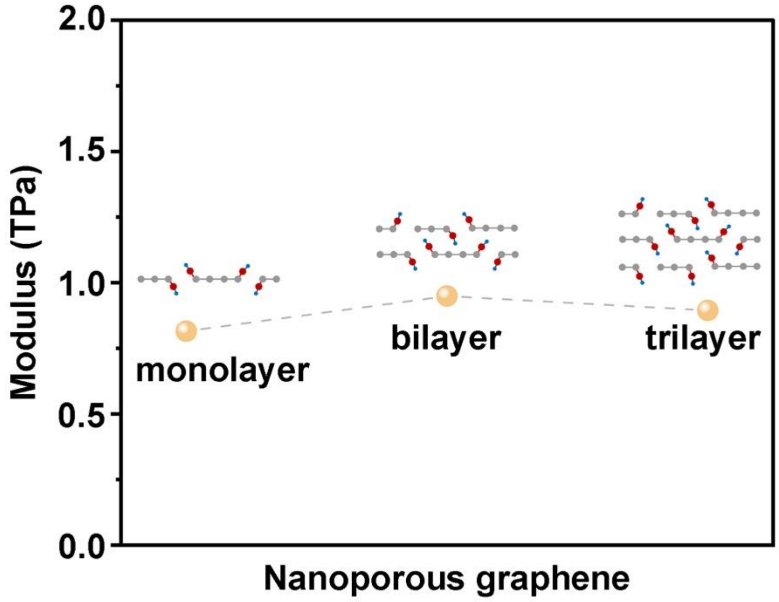

Figure S13. Young's modulus of monolayer graphene nanomesh, and SNGMs assembled by bilayer and trilayer graphene nanomesh in MD simulations.

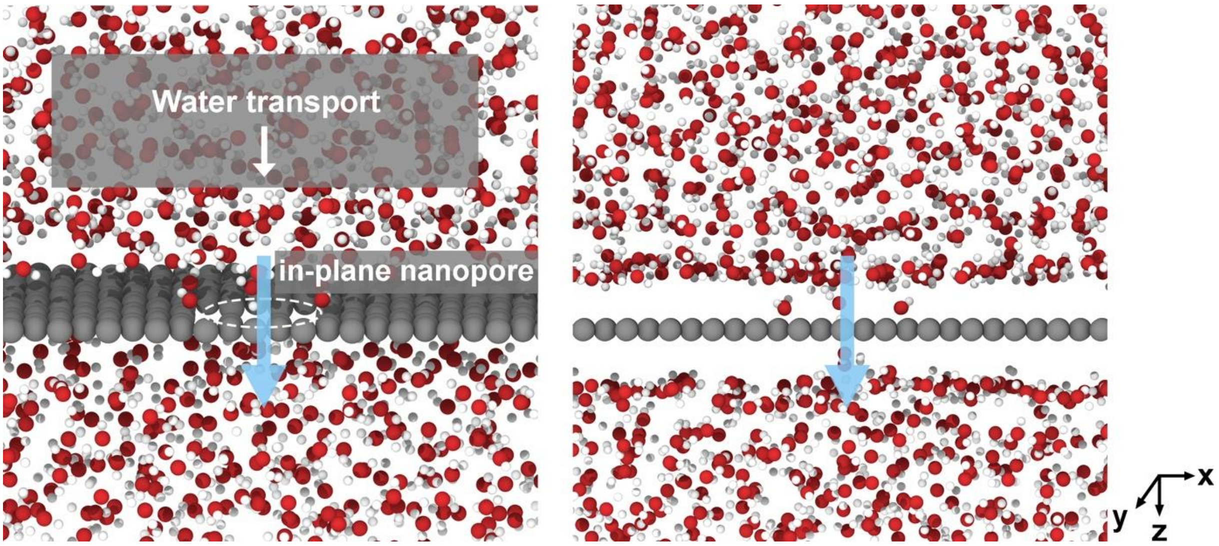

Figure S14. A snapshot of the water transport in the monolayer graphene nanomesh.

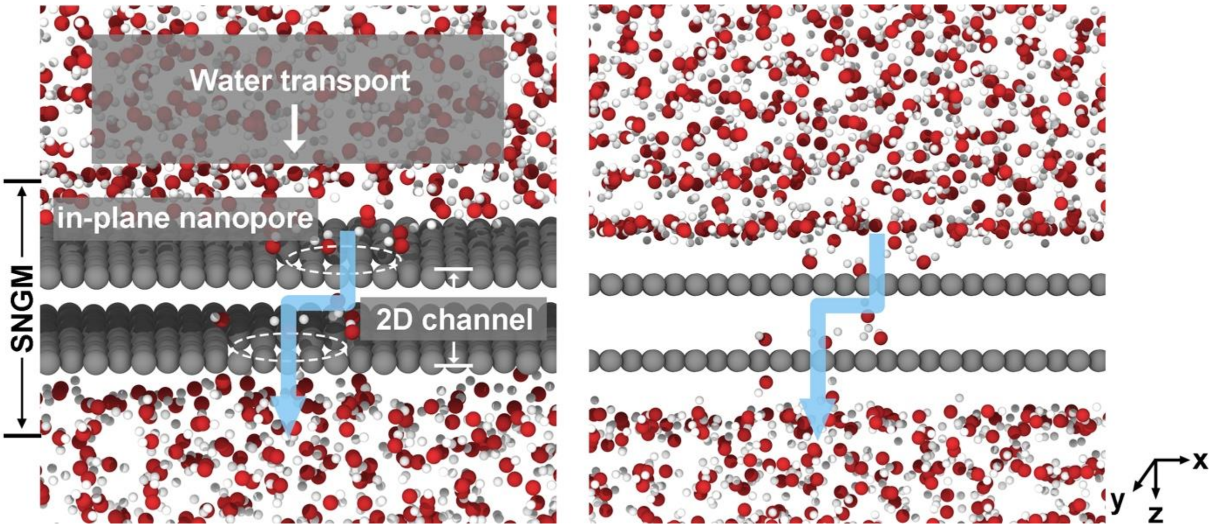

Figure S15. A snapshot of the water transport in the SNGM assembled by bilayer graphene nanomesh.

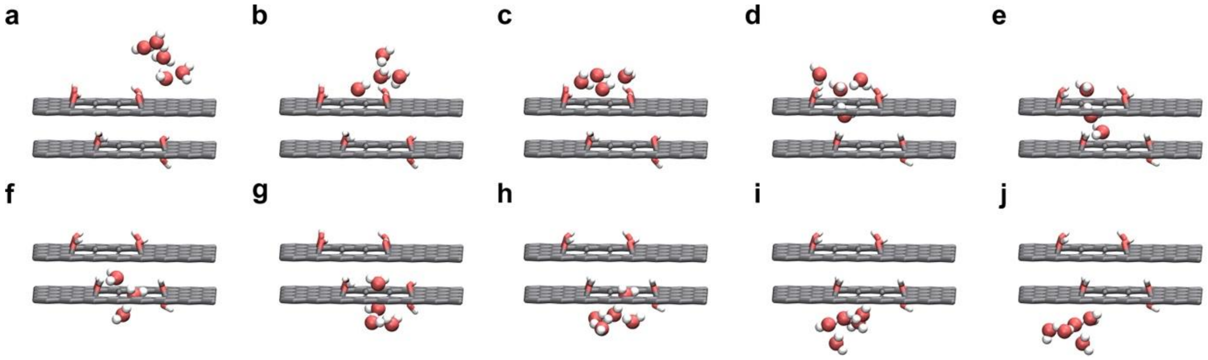

Figure S16. A snapshot of water molecules transport processes in the in-plane nanopores and 2D nanochannels of the SNGMs.

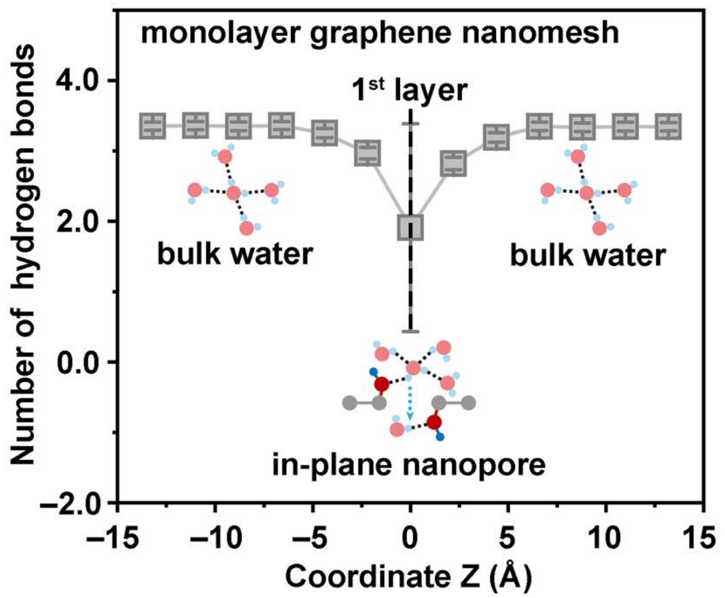

Figure S17. Hydroxy bonds distribution among water molecules in the monolayer graphene nanomesh.

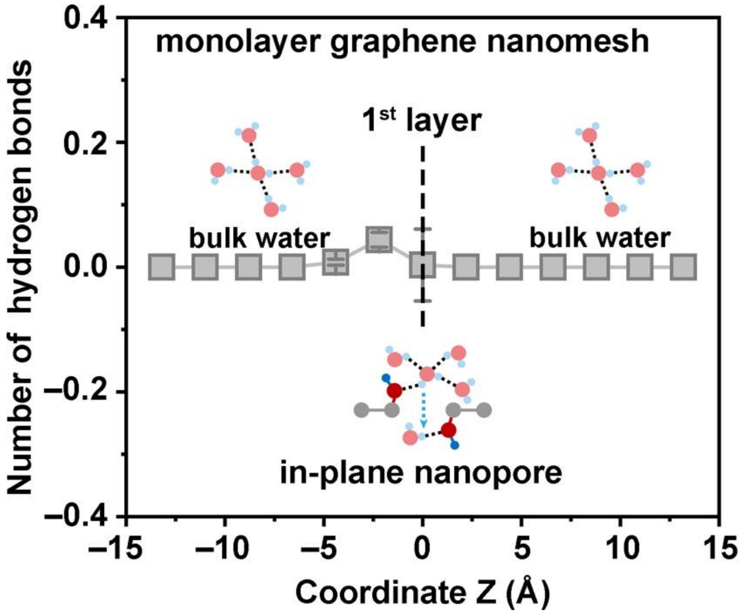

Figure S18. Hydroxy bonds distribution between water molecules and the monolayer graphene nanomesh.

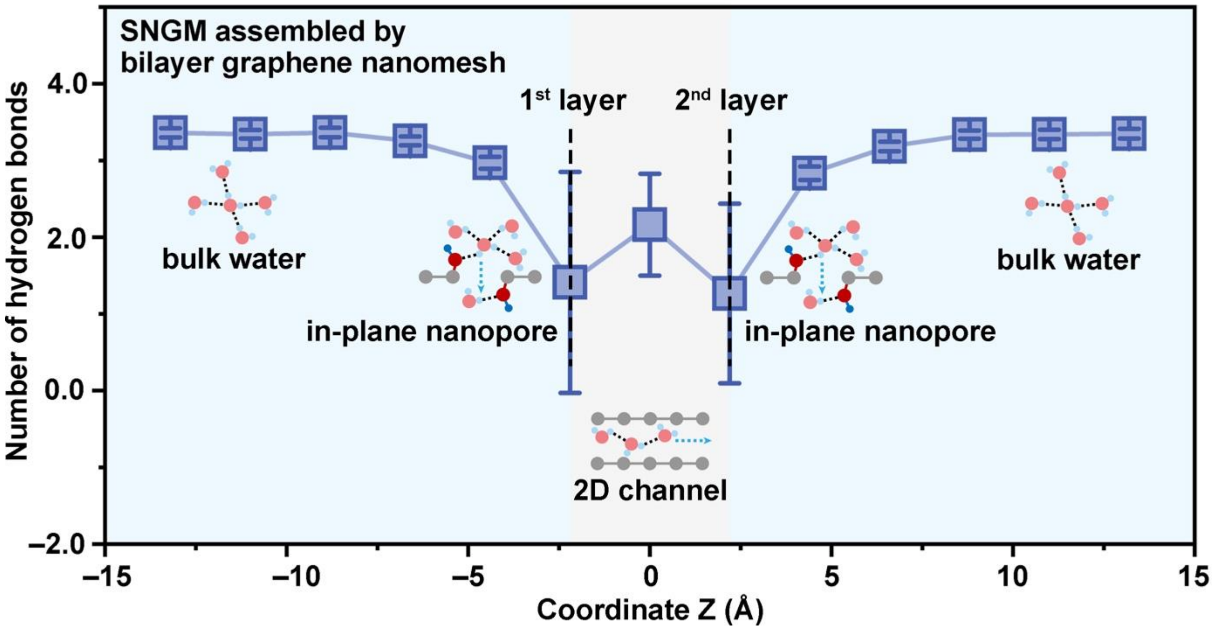

Figure S19. Hydroxy bonds distribution among water molecules in the SNGM assembled by bilayer graphene nanomesh.

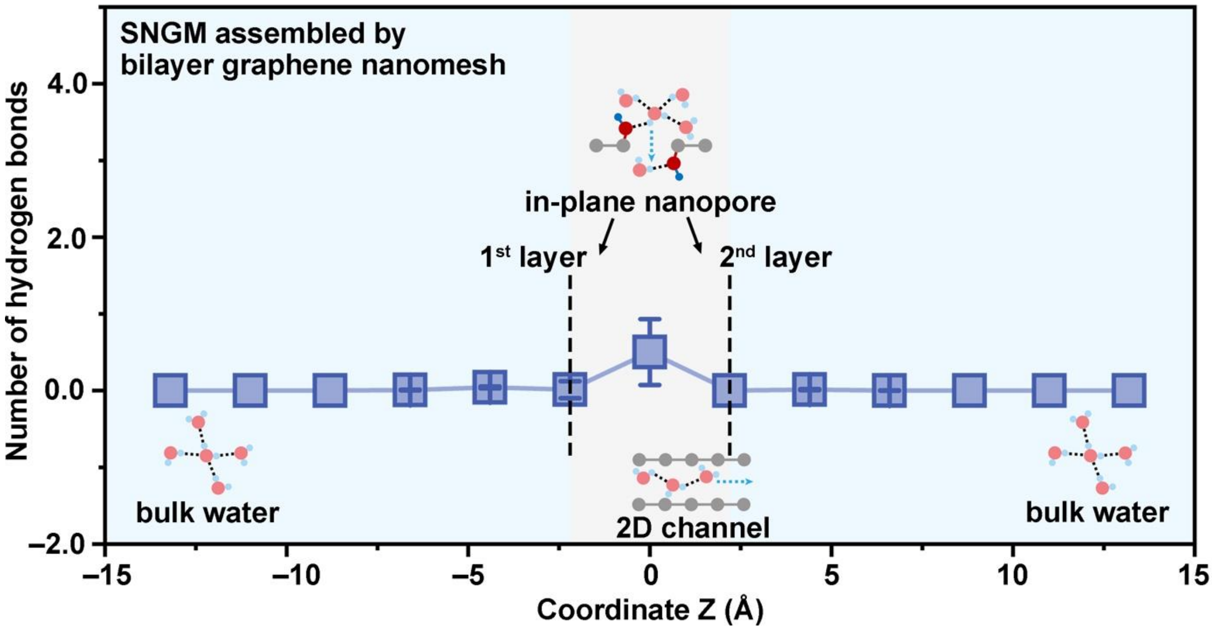

Figure S20. Hydroxy bonds distribution between water molecules and the SNGM assembled by bilayer graphene nanomesh.

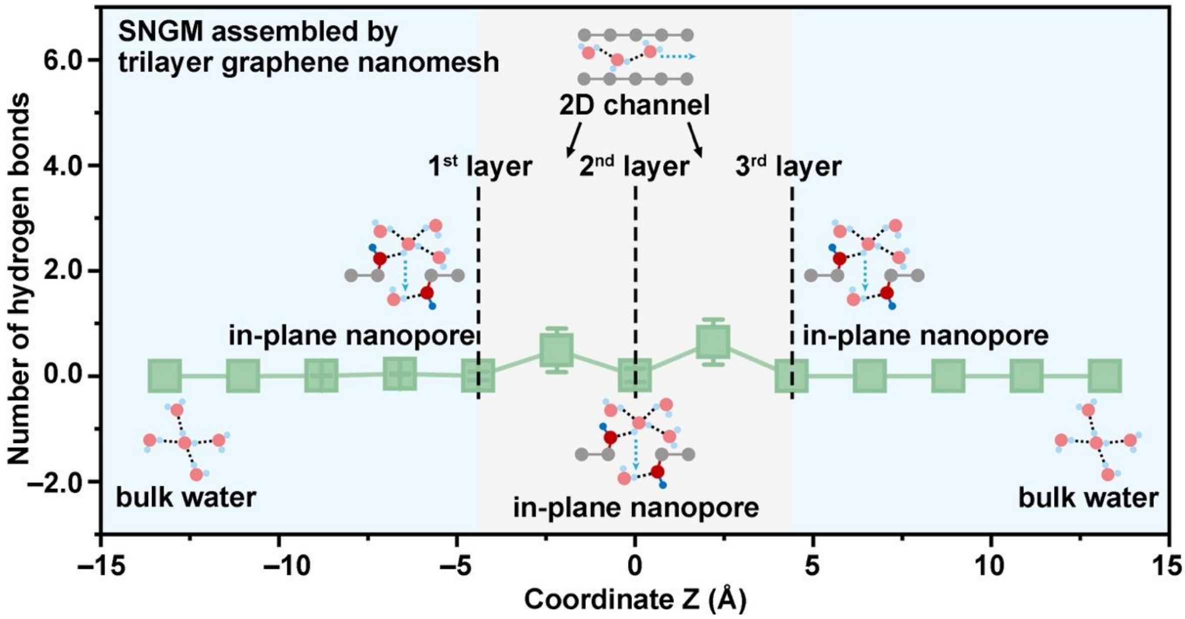

Figure S21. Hydroxy bonds distribution between water molecules and the SNGM assembled by trilayer graphene nanomesh.

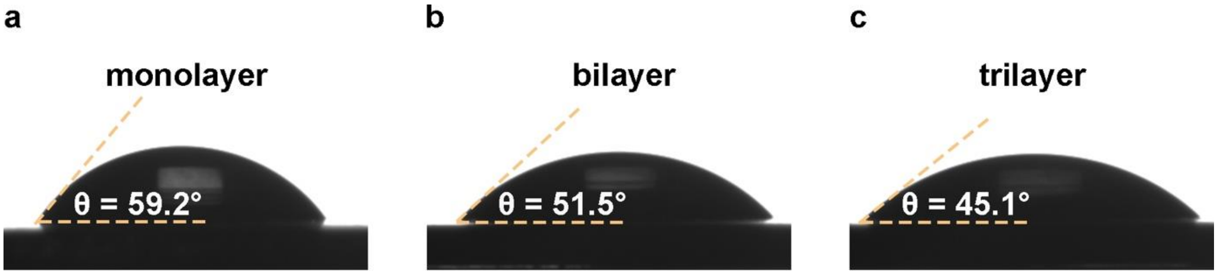

Figure S22. Contact angle of the SNGMs. (a) monolayer graphene nanomesh. (b) SNGM assembled by bilayer graphene nanomesh and (c) SNGM assembled by trilayer graphene nanomesh.

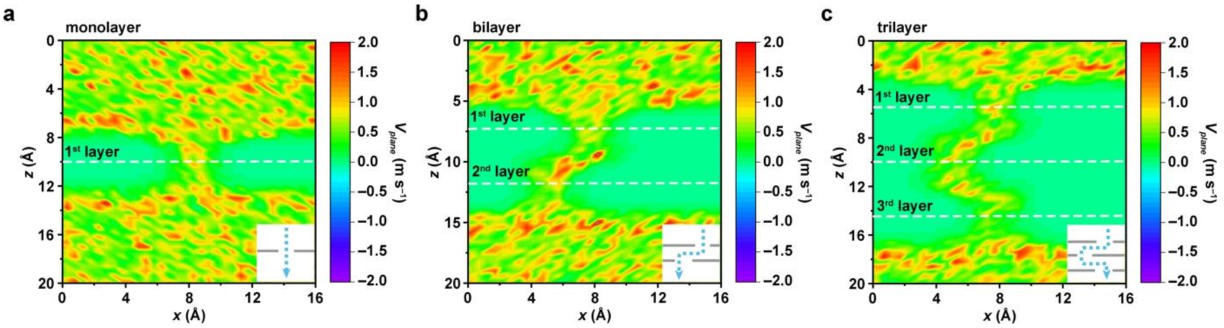

Figure S23. Time-averaged water molecules transport velocity in the monolayer graphene nanomesh, SNGMs assembled by bilayer and trilayer graphene nanomesh (x-y plane). (a) monolayer graphene nanomesh. (b) SNGM assembled by bilayer graphene nanomesh and (c) SNGM assembled by trilayer graphene nanomesh.

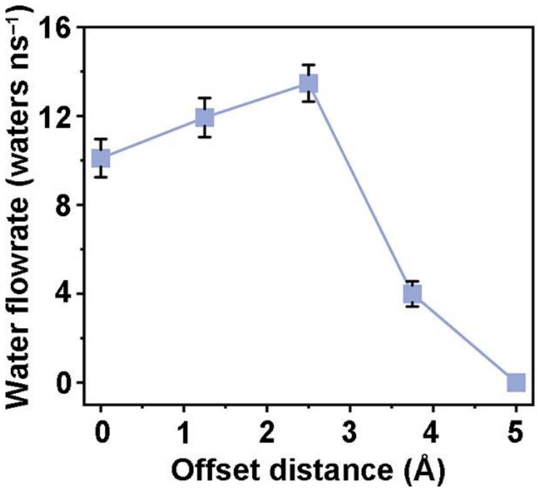

Figure S24. Effect of pore offset of membrane configurational parameters on the water flowrate in the SNGM assembled by bilayer graphene nanomesh.

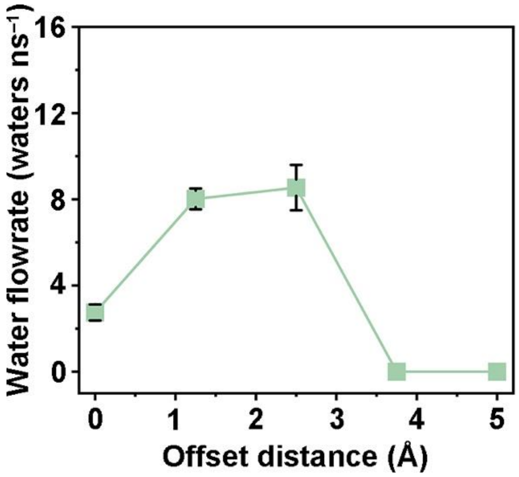

Figure S25. Effect of pore offset of membrane configurational parameters on the water flowrate in the SNGM assembled by trilayer graphene nanomesh.

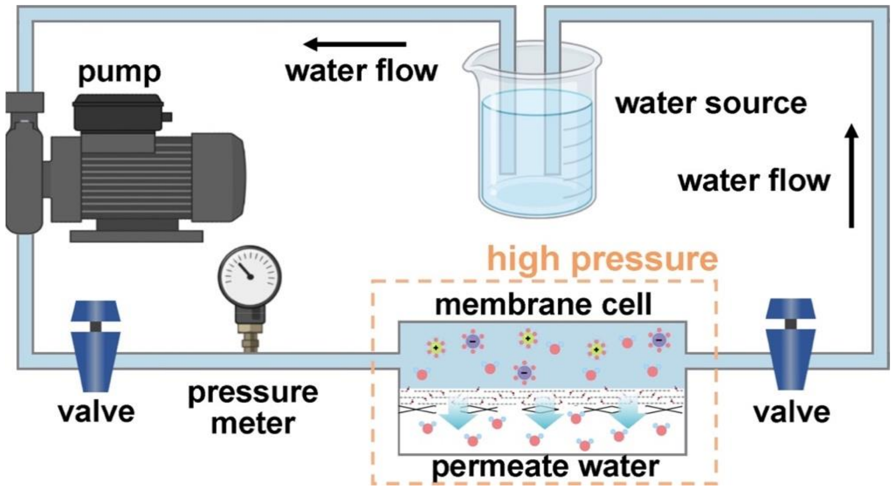

Figure S26. Schematic illustration of the RO cross-flow filtration apparatus.

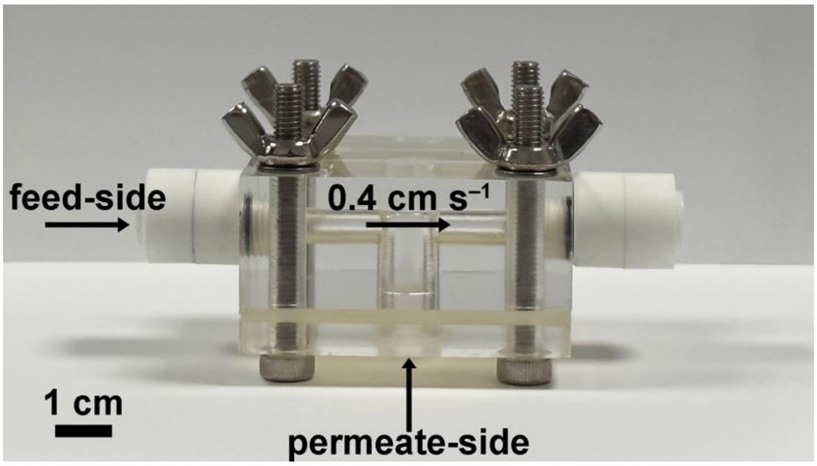

Figure S27. Photograph of the membrane cell in the RO cross-flow filtration apparatus.

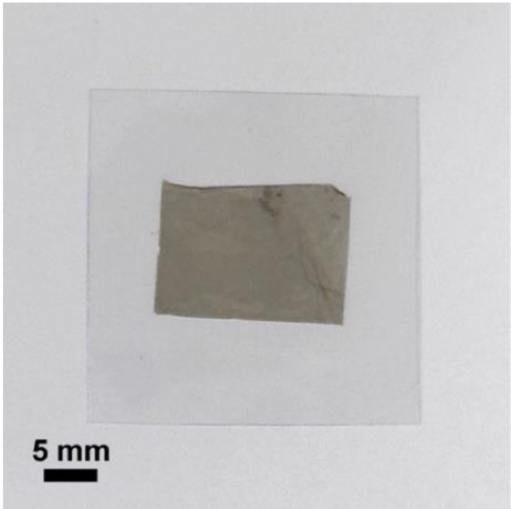

Figure S28. Photograph of the SNGM assembled by trilayer graphene nanomesh supported by SWNT suspended on a PET substrate.

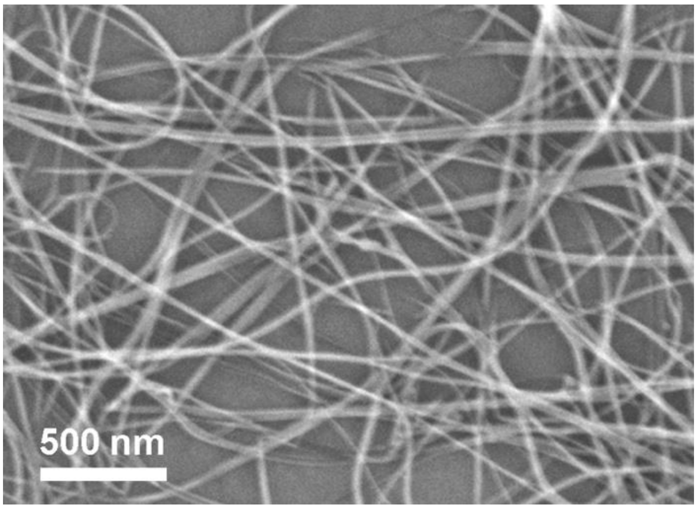

Figure S29. SEM image of the SNGM assembled by trilayer graphene nanomesh supported by SWNT.

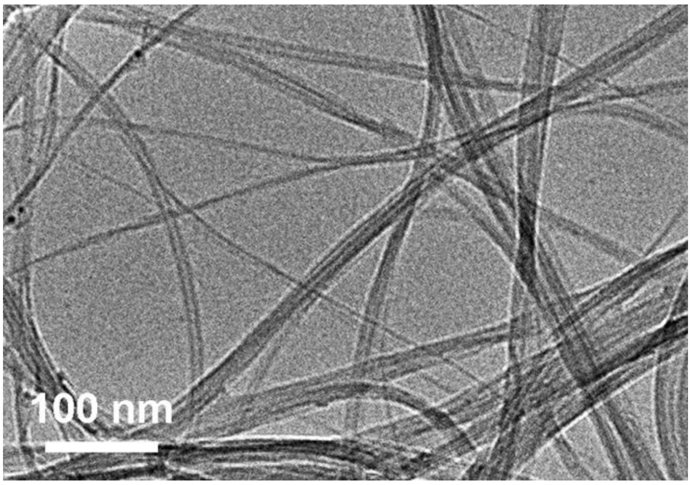

Figure S30. TEM image of the SNGM assembled by trilayer graphene nanomesh supported by SWNT.

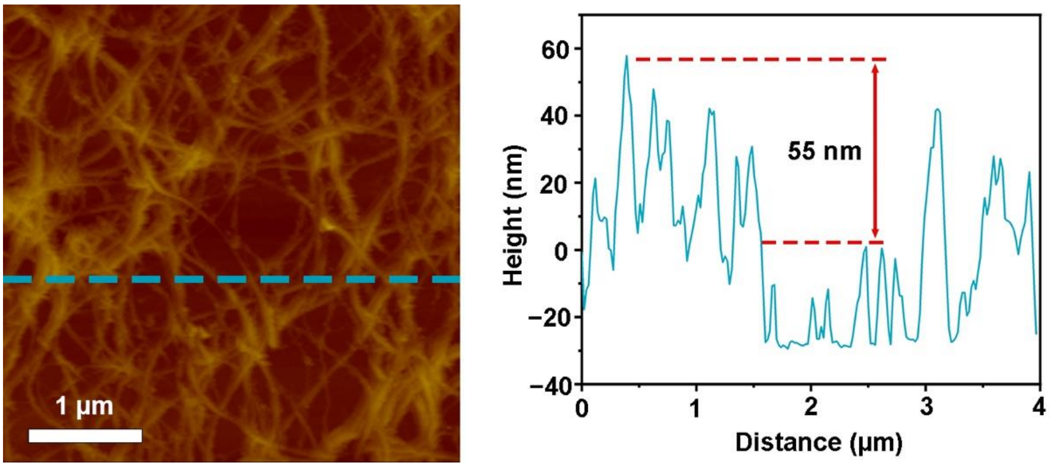

Figure S31. AFM image and height profile of the SNGM assembled by trilayer graphene nanomesh supported by SWNT.

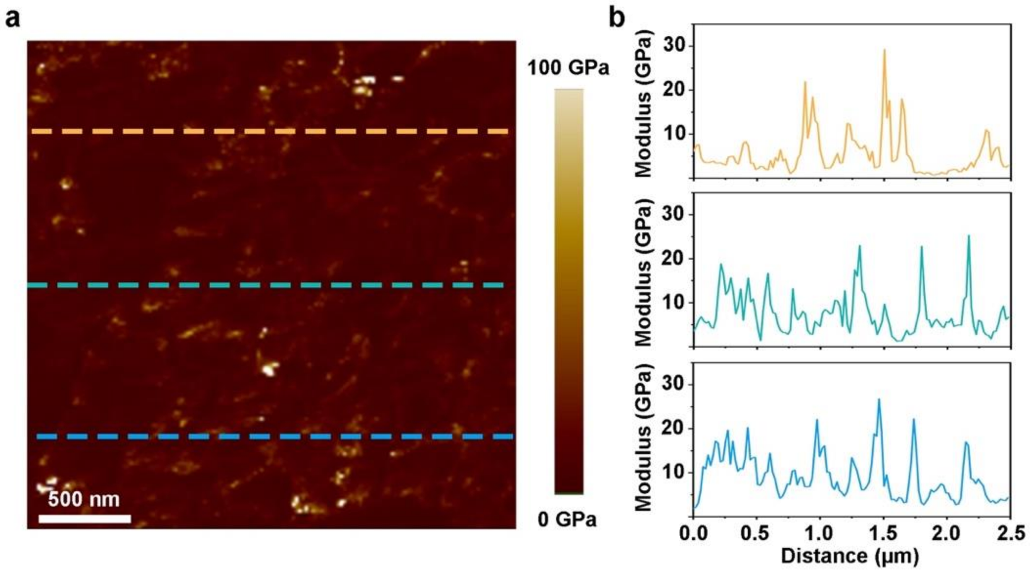

Figure S32. Modulus map and modulus distribution of SNGM assembled by trilayer graphene nanomesh supported by SWNT.

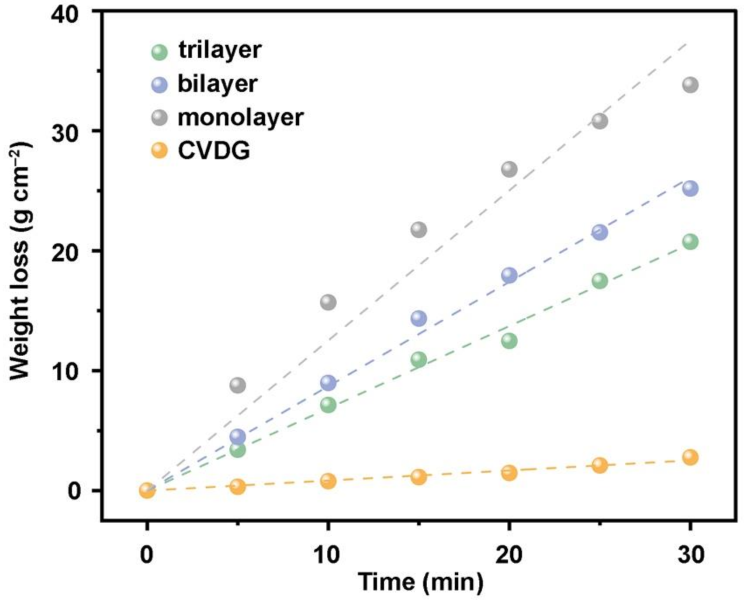

Figure S33. Water permeation performance of the SNGMs. Water permeation through the monolayer graphene nanomesh, SNGMs assembled by bilayer, and trilayer graphene nanomesh prepared by O_2 plasma etching time of 10 s.

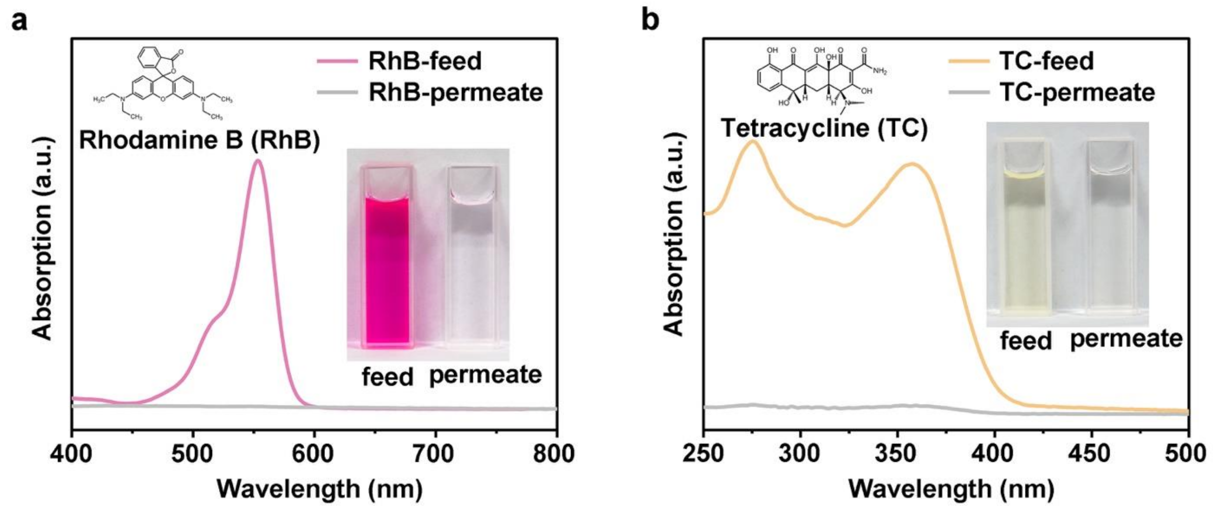

Figure S34. Normalized absorption spectra of the dye molecules (Rhodamine B, RhB) and antibiotics (tetracycline, TC) before and after filtration.

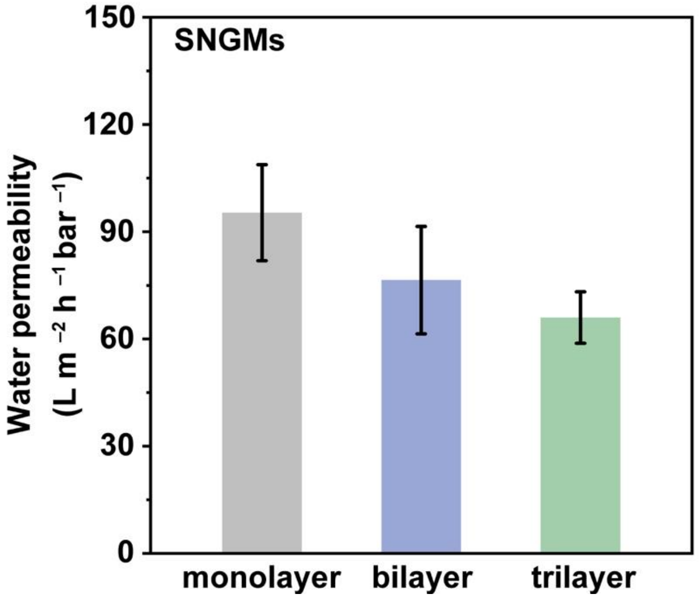

Figure S35. Water permeability of monolayer graphene nanomesh, and SNGMs assembled by bilayer and trilayer graphene nanomesh for 2000 ppm NaCl solution. Error bars indicate the standard deviation from three different samples.

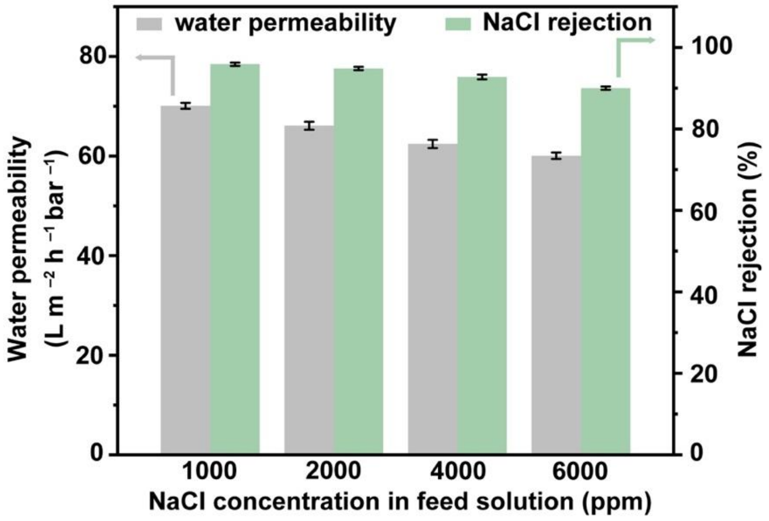

Figure S36. Reverse osmosis separation performance. Water permeability and salt rejection of the SNGM assembled by trilayer graphene nanomesh. Different concentrations of NaCl were used as the feed solution. The vertical lines present standard deviations acquired from three individual membranes.

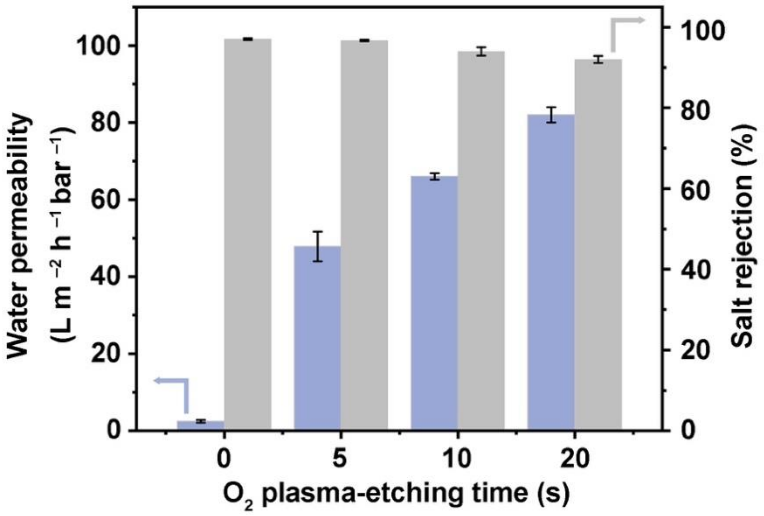

Figure S37. Water permeability and salt rejection of the SNGM assembled by trilayer graphene nanomesh with O_2 plasma-etching time of 5, 10, and 20 s, respectively. The vertical lines present standard deviations acquired from three individual membranes. NaCl solution with a concentration of 2000 ppm was used as the feed solution.

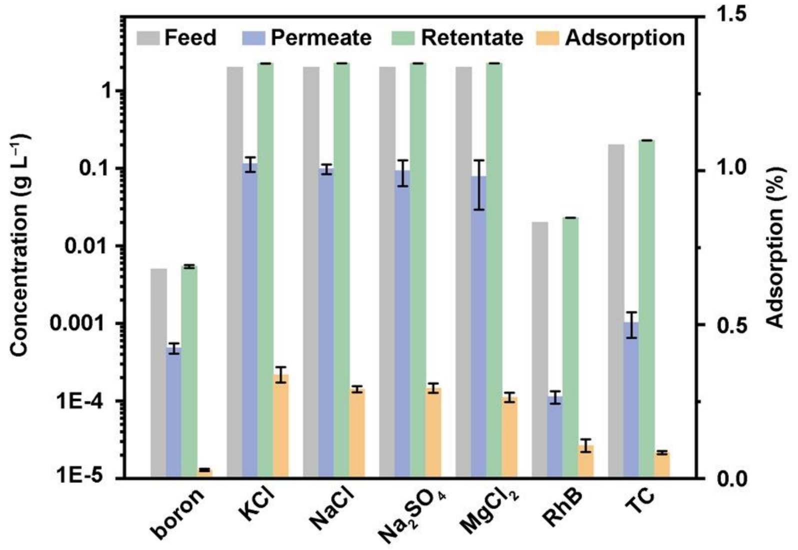

Figure S38. Analysis of the adsorption percentage of SNGM assembled by trilayer graphene nanomesh for salt, dye molecules and antibiotic during the filtration tests. Error bars indicate the standard deviations from three different membranes.

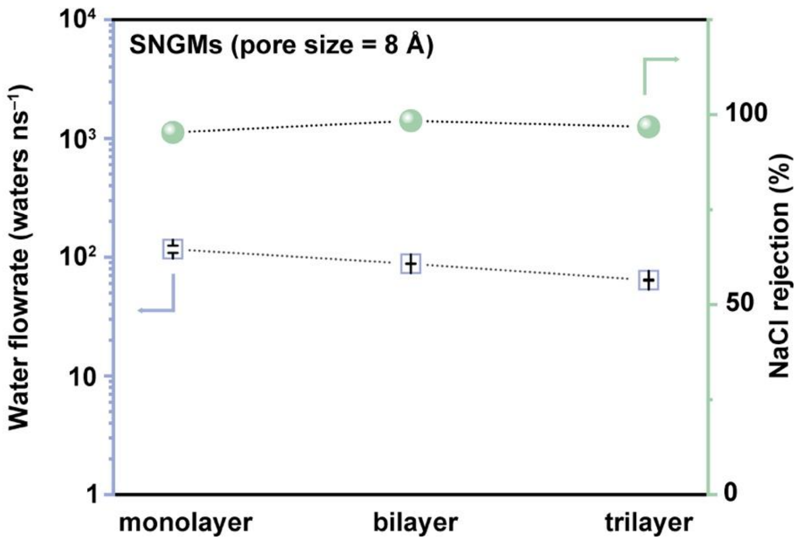

Figure S39. The calculated water flowrate in NaCl solutions and NaCl rejection of the SNGMs with the pore size of 8 Å in MD simulations.

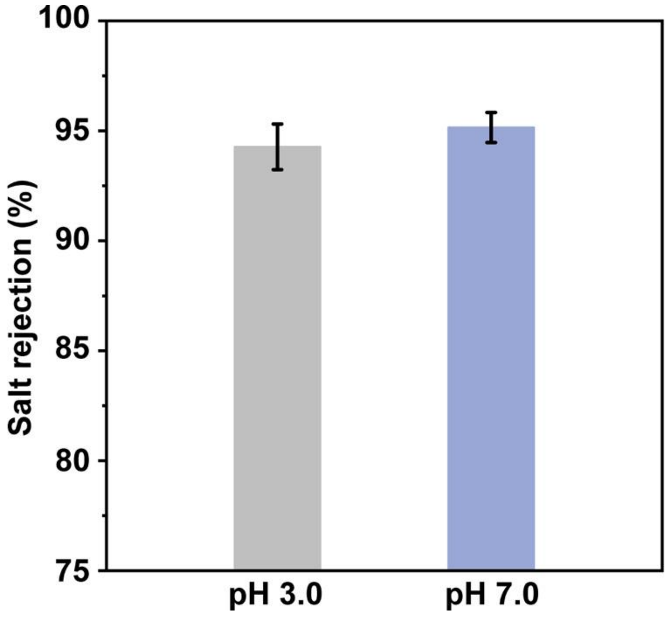

Figure S40. Salt rejection of the SNGM assembled by trilayer graphene nanomesh measured at pH values of 3.0 and 7.0 for 2000 ppm NaCl solution. The vertical lines present standard deviations acquired from three individual membranes.

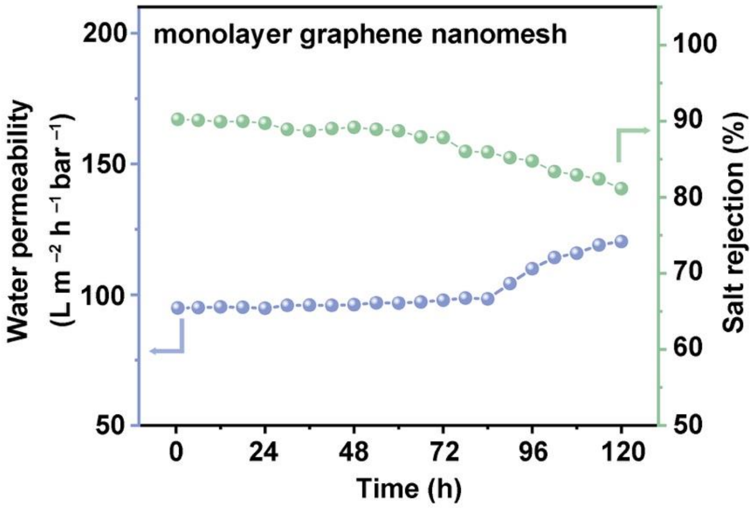

Figure S41. Long-term stability of the monolayer graphene nanomesh.

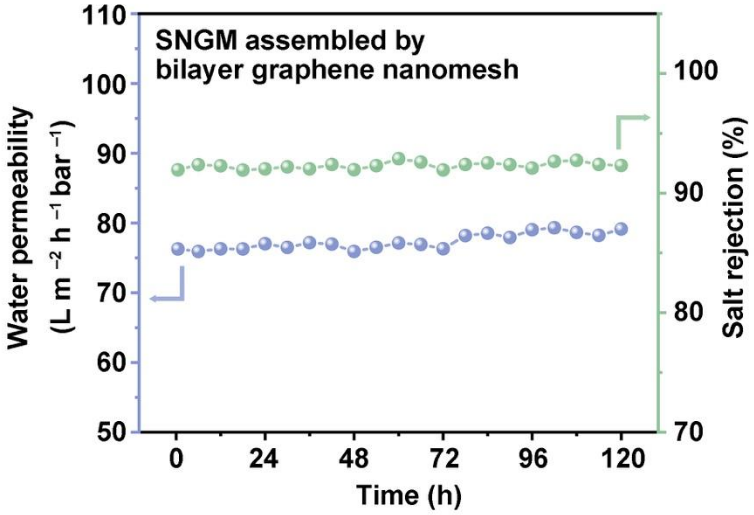

Figure S42. Long-term stability of the SNGM assembled by bilayer graphene nanomesh.

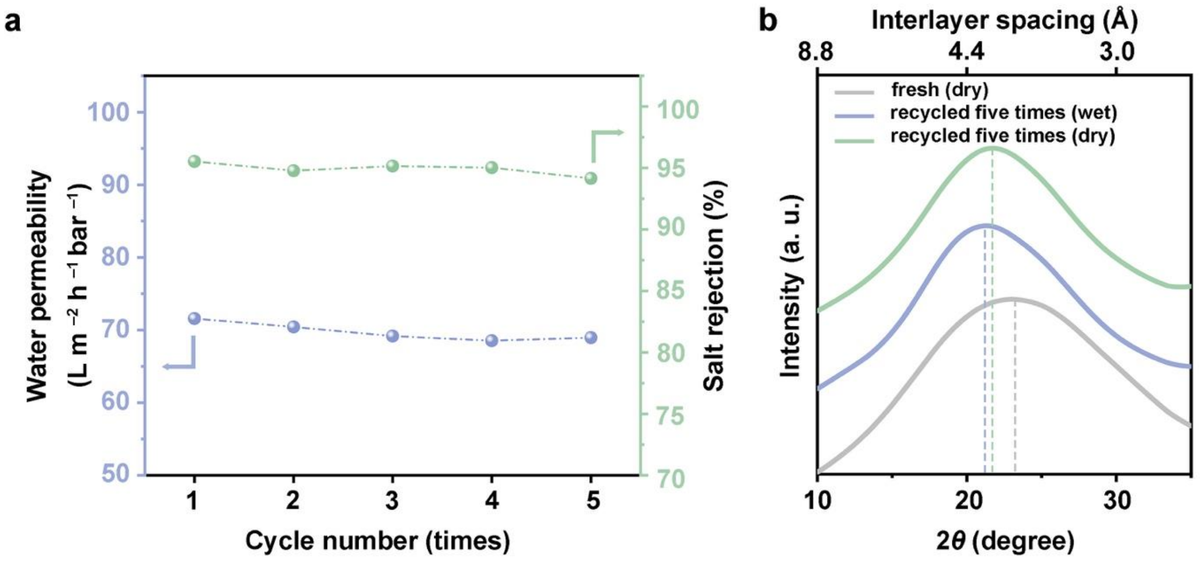

Figure S43. Cycle performance of the SNGM assembled by trilayer graphene nanomesh. (a) Water permeability and salt rejection of the SNGM assembled by trilayer graphene nanomesh for NaCl solutions after five cycles. (b) XRD results and the interlayer spacing of the SNGM assembled by trilayer graphene nanomesh in the dry state and after five cycles of desalination/drying.

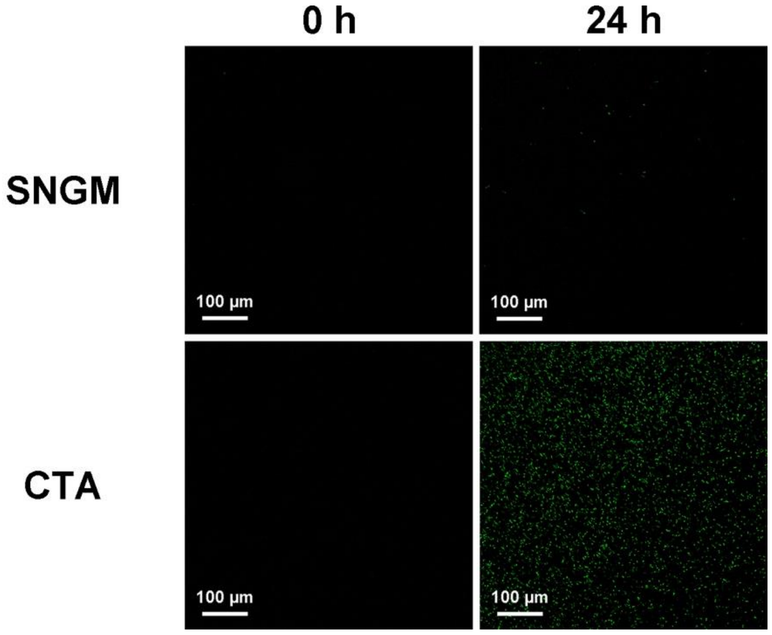

Figure S44. Fluorescence microscopy images of *S. putrefaciens* cells on the SNGM assembled by trilayer graphene nanomesh and CTA membrane after 24 h of operation.

Supplementary Table

Table S1. XPS survey for CVDG, monolayer graphene nanomesh, SNGMs assembled by bilayer and trilayer graphene nanomesh.

CVDG				
Element	Peak position (eV)	FWHM (eV)	Area	At.%
C	284	1.62	99600	25.93
O	532	1.83	248028	30.99
monolayer graphene nanomesh				
Element	Peak position (eV)	FWHM (eV)	Area	At.%
C	284	1.11	119870	52.13
O	532	1.73	140162	16.21
SNGMs assembled by bilayer graphene nanomesh				
Element	Peak position (eV)	FWHM (eV)	Area	At.%
C	284	1.38	147457	49.25
O	532	2.06	219417	24.33
SNGMs assembled by trilayer graphene nanomesh				
Element	Peak position (eV)	FWHM (eV)	Area	At.%
C	284	1.33	167412	53.18
O	532	2.17	208584	19.81

Table S2. Comparison of the water permeability and salt rejection performance of the SNGM with state-of-the-art membranes reported.

Membrane	Feed solution	Salt rejection (%)	NaCl solution water permeability ($L\ m^{-2}\ h^{-1}\ bar^{-1}$)	Pure water permeability ($L\ m^{-2}\ h^{-1}\ bar^{-1}$)	References
GO	1170 ppm NaCl	19		27.6	[13]
rGO	1170 ppm NaCl	42		3.3	[14]
GO-CNTs	584.4 ppm NaCl	59	5.5		[15]
GO-TBO	584.4 ppm NaCl	81	0.4	0.41	[16]
Shear-aligned GO	2000 ppm NaCl	33		71	[4]
SNGM	2000 ppm NaCl	95.2	66.0	77.8	This work
MOF-303	1000 ppm NaCl	33.2	0.74		[17]
ZIF-8@f-Gom	1000 ppm NaCl	30.3	49.8	60	[18]
UIO-66	2000 ppm NaCl	50	0.14		[19]
TpPa-COF	1000 ppm NaCl	49.2	60		[20]
sCPF	584.4 ppm NaCl	83	9.3	9.5	[3]
BW30	2000 ppm NaCl	98.9	3.47		[21]
SW30	2000 ppm NaCl	93.8	0.7		[22]
X20	2000 ppm NaCl	98.7	2.87		[22]
GE AG	2000 ppm NaCl	90.2	2.45		[22]
Synder NFX	2000 ppm	40		2.4	[23]

	NaCl				
Synder NFW	2000 ppm NaCl	20		5.4	[23]
Synder NF 2A	2000 ppm NaCl	24.8		10.1	[24]

Supplementary Video

Video S1. Water molecules transport processes in the in-plane nanopores and 2D nanochannels of the SNGMs.

References

1. Yang Q, Su Y, Chi C *et al.* Ultrathin graphene-based membrane with precise molecular sieving and ultrafast solvent permeation. *Nat Mater* 2017; **16**: 1198–1202.
2. Sun Y, Yi F, Li R *et al.* Inorganic-Organic Hybrid Membrane Based on Pillararene-Intercalated MXene Nanosheets for Efficient Water Purification. *Angew. Chem. Int. Ed.* 2022; **61**: e202200482.
3. Shen J, Cai Y, Zhang C *et al.* Fast water transport and molecular sieving through ultrathin ordered conjugated-polymer-framework membranes. *Nat Mater* 2022; **21**: 1183–1190.
4. Akbari A, Sheath P, Martin S *et al.* Large-area graphene-based nanofiltration membranes by shear alignment of discotic nematic liquid crystals of graphene oxide. *Nat Commun* 2016; **7**: 10891.
5. Plimpton, S. FAST PARALLEL ALGORITHMS FOR SHORT-RANGE MOLECULAR-DYNAMICS. *J Comput Phys* 1995; **117**: 1–19.
6. Ghorbanzadeh Ahangari M, Salmankhani A, Imani A *et al.* Density functional theory study on the mechanical properties and interlayer interactions of multi-layer graphene: carbonic, silicon-carbide and silicene graphene-like structures. *Silicon* 2019; **11**: 1235–1246.
7. Han J, Ryu S, Kim D *et al.* Effect of interlayer sliding on the estimation of elastic modulus of multilayer graphene in nanoindentation simulation. *Europhys Lett* 2016; **114**: 68001.
8. Stuart S, Tutein A, Harrison J. A reactive potential for hydrocarbons with intermolecular interactions. *J Chem Phys* 2000; **112**: 6472–6486.
9. Downs R, Terdalkar S, Rencis J. NANO-INDENTATION OF MONOLAYER GRAPHENE SHEET USING MOLECULAR DYNAMIC SIMULATIONS. In *2009 ASME Early Career Technical Conference*, 2009.
10. Joung I, Cheatham T. III. Determination of alkali and halide monovalent ion parameters for use in explicitly solvated biomolecular simulations. *J Phys Chem B* 2008; **112**: 9020–9041.
11. Beu T. Molecular dynamics simulations of ion transport through carbon nanotubes. I. Influence of geometry, ion specificity, and many-body interactions. *J Chem Phys* 2010; **132**: 164513.
12. Mooney D, Muller-Plathe F, Kremer K. Simulation studies for liquid phenol: properties evaluated and tested over a range of temperatures. *Chem Phys Lett* 1998; **294**: 135–142.
13. Hu M, Mi B. Enabling Graphene Oxide Nanosheets as Water Separation Membranes. *Environ Sci Technol* 2013; **47**: 3715–3723.
14. Han Y, Xu Z, Gao C. Ultrathin Graphene Nanofiltration Membrane for Water Purification. *Adv Funct Mater* 2013; **23**: 3693–3700.
15. Han Y, Jiang Y, Gao C. High-Flux Graphene Oxide Nanofiltration Membrane Intercalated by Carbon Nanotubes. *ACS Appl Mater Interfaces* 2015; **7**: 8147–8155.
16. Wang Z, Ma C, Xu C *et al.* Graphene oxide nanofiltration membranes for desalination under realistic conditions. *Nat Sustain* 2021; **4**: 402–408.
17. Cong S, Yuan Y, Wang J *et al.* Highly Water-Permeable Metal-Organic Framework MOF-303 Membranes for Desalination. *J Am Chem Soc* 2021; **143**: 20055–20058.
18. Zhang W, Yin M, Zhao Q *et al.* Graphene oxide membranes with stable porous structure for ultrafast water transport. *Nat Nanotechnol* 2021; **16**: 337–343.
19. Liu X, Demir N, Wu Z *et al.* Highly Water-Stable Zirconium Metal Organic Framework UiO-66 Membranes Supported on Alumina Hollow Fibers for Desalination. *J Am Chem Soc* 2015; **137**: 6999–7002.
20. Zhang Y, Guo J, Han G *et al.* Molecularly soldered covalent organic frameworks for

ultrafast precision sieving. *Sci Adv* 2021; **7**: eabe8706.
21. Choi W, Jeon S, Kwon S *et al.* Thin film composite reverse osmosis membranes prepared via layered interfacial polymerization. *J Membr Sci* 2017; **527**: 121–128.
22. Jiang Z, Karan S, Livingston A. Water Transport through Ultrathin Polyamide Nanofilms Used for Reverse Osmosis. *Adv Mater* 2018; **30**: 1705973.
23. Tang Y, Xu Z, Xue S *et al.* A chlorine-tolerant nanofiltration membrane prepared by the mixed diamine monomers of PIP and BHTTM. *J Membr Sci* 2016; **498**: 374–384.
24. Lin J, Tang C, Huang C *et al.* A comprehensive physico-chemical characterization of superhydrophilic loose nanofiltration membranes. *J Membr Sci* 2016; **501**: 1–14.
